# Supplementary material for: Pandemic velocity: Forecasting COVID-19 in the US with a machine learning & Bayesian time series compartmental model
Source: PLoS Comput Biol. 2021 Mar 29;17(3):e1008837. doi: 10.1371/journal.pcbi.1008837 (PMC8031749; doi:10.1371/journal.pcbi.1008837)
Supplement: S1 Appendix — The derivation of the compartmental model transition function from the autoregressive velocity model. (PDF) [file pcbi.1008837.s001.pdf]

Pandemic velocity: forecasting COVID-19 in the U.S. with a machine learning & Bayesian time series compartmental model, supplemental material

Gregory L. Watson[1], Di Xiong[1], Lu Zhang[1], Joseph A. Zoller[1], John Shamshoian[1], Phillip Sundin[1], Teresa Bufford[1], Anne W. Rimoin[2], Marc A. Suchard[1,3], Christina M. Ramirez[1]

1 Department of Biostatistics, Fielding School of Public Health, University of California, Los Angeles, California, United States of America
2 Department of Epidemiology, Fielding School of Public Health, University of California, Los Angeles, California, United States of America
3 Departments of Computational Medicine and Human Genetics, David Geffen School of Medicine, University of California, Los Angeles, California, United States of America

S1 Appendix. Derivation of Case Transition Function

$$\begin{aligned}
\log y_i(t) &= \mu_i + \phi_i \log y_i(t-1) + \epsilon_i(t) \\
\log y_i(t) - \phi_i \log y_i(t-1) &= \mu_i + \epsilon_i(t) \\
\log \frac{y_i(t)}{y_i(t-1)^{\phi_i}} &= \mu_i + \epsilon_i(t) \\
\frac{y_i(t)}{y_i(t-1)^{\phi_i}} &= \exp\left[\mu_i + \epsilon_i(t)\right]
\end{aligned}$$

To avoid stochastic differential equations in the compartmental model, we replace the right hand side $\exp\left[\mu_i + \epsilon_i(t)\right]$ with its expectation, $E \exp\left[\mu_i + \epsilon_i(t)\right] = \exp\left[\mu_i + \frac{1}{2\tau_i}\right]$, yielding

$$\begin{aligned}
\frac{y_i(t)}{y_i(t-1)^{\phi_i}} &:= \exp\left[\mu_i + \frac{1}{2\tau_i}\right] \\
y_i(t) &= y_i(t-1)^{\phi_i} \exp\left[\mu_i + \frac{1}{2\tau_i}\right] \\
\frac{d \log u_i(t)}{dt} &= \left[\frac{d \log u_i(t-1)}{dt}\right]^{\phi_i} \exp\left[\mu_i + \frac{1}{2\tau_i}\right] \\
\frac{\frac{d}{dt} u_i(t)}{u_i(t)} &= \left[\frac{\frac{d}{dt} u_i(t-1)}{u_i(t-1)}\right]^{\phi_i} \exp\left[\mu_i + \frac{1}{2\tau_i}\right] \\
\frac{d}{dt} u_i(t) &= u_i(t) \left[\frac{\frac{d}{dt} u_i(t-1)}{u_i(t-1)}\right]^{\phi_i} \exp\left[\mu_i + \frac{1}{2\tau_i}\right]
\end{aligned}$$

This expression assumes an infinite pool of susceptible persons, and so we apply an adjustment by dividing $S_i(t)$ by the number of susceptible persons at time t_0,

$$\frac{d}{dt}u_i(t) \coloneqq u_i(t)\left[\frac{\frac{d}{dt}u_i(t-1)}{u_i(t-1)}\right]^{\phi_i}\exp\left[\mu_i + \frac{1}{2\tau_i}\right]\frac{S_i(t)}{S_i(t_0)}.$$
