# Supplementary material for: Pandemic velocity: Forecasting COVID-19 in the US with a machine learning & Bayesian time series compartmental model
Source: PLoS Comput Biol. 2021 Mar 29;17(3):e1008837. doi: 10.1371/journal.pcbi.1008837 (PMC8031749; doi:10.1371/journal.pcbi.1008837)
Supplement: S2 Appendix — Projected cumulative case count, active confirmed infections, and daily deaths through April 1, 2021, for each of the 50 U.S. states. (PDF) [file pcbi.1008837.s002.pdf]

AL

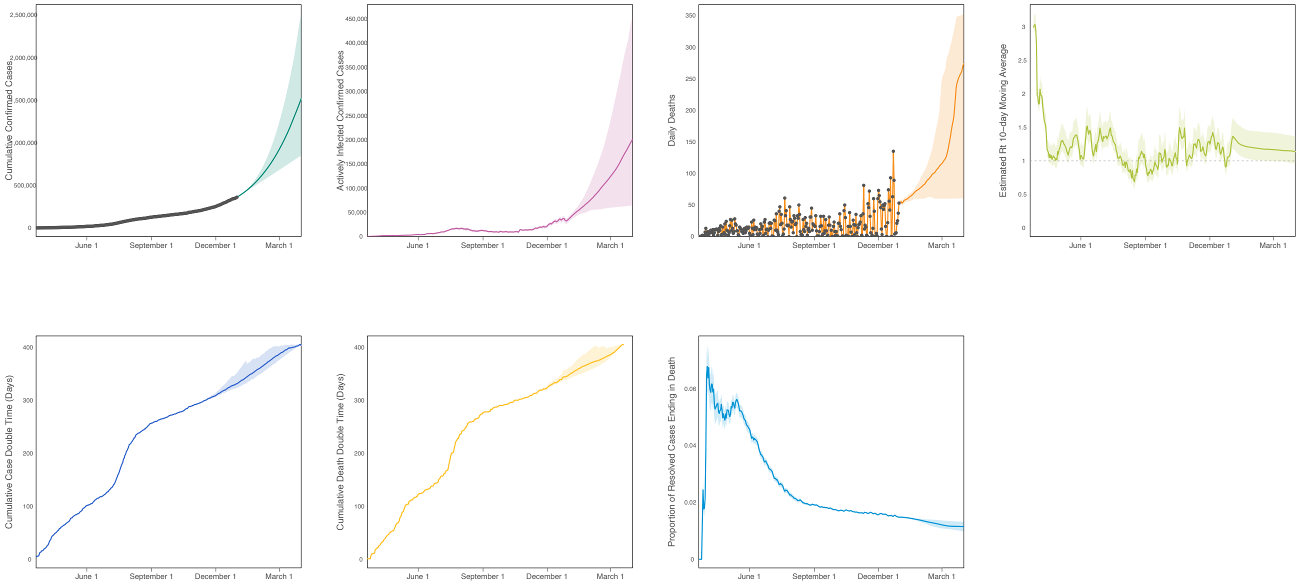

Cumulative Confirmed Cases
Actively Infected Confirmed Cases
Daily Deaths
Estimated Rt 10-day Moving Average
Cumulative Case Double Time (Days)
Cumulative Death Double Time (Days)
Proportion of Resolved Cases Ending in Death
June 1
September 1
December 1
March 1

AK

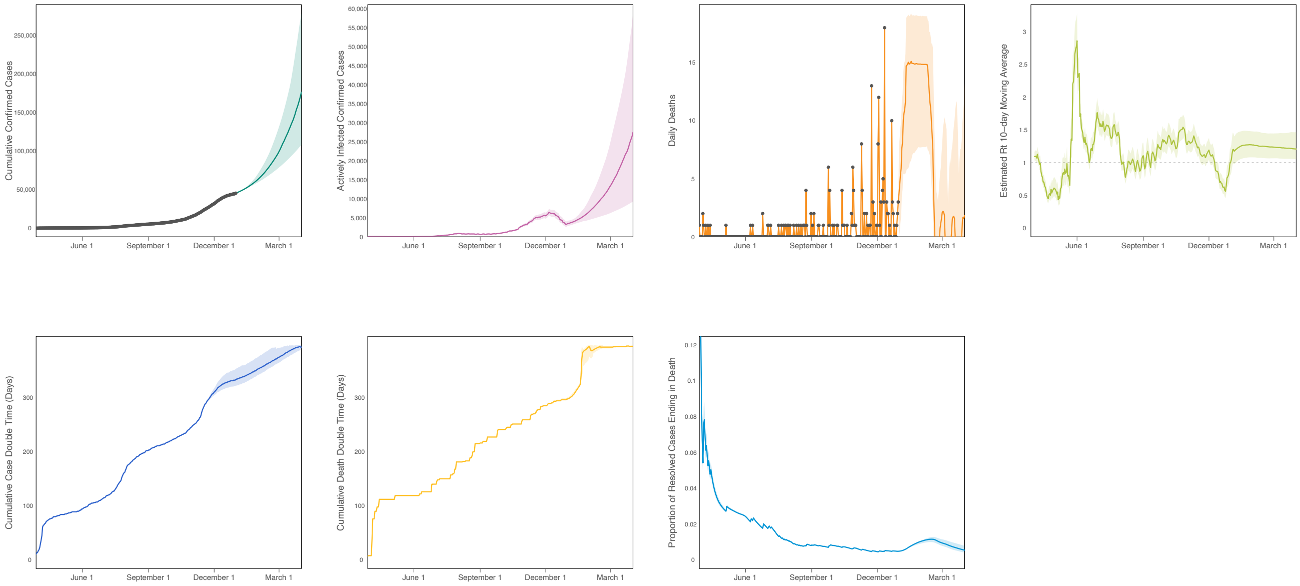

Cumulative Confirmed Cases
Actively Infected Confirmed Cases
Daily Deaths
Estimated Rt 10-day Moving Average
Cumulative Case Double Time (Days)
Cumulative Death Double Time (Days)
Proportion of Resolved Cases Ending in Death
June 1
September 1
December 1
March 1

AZ

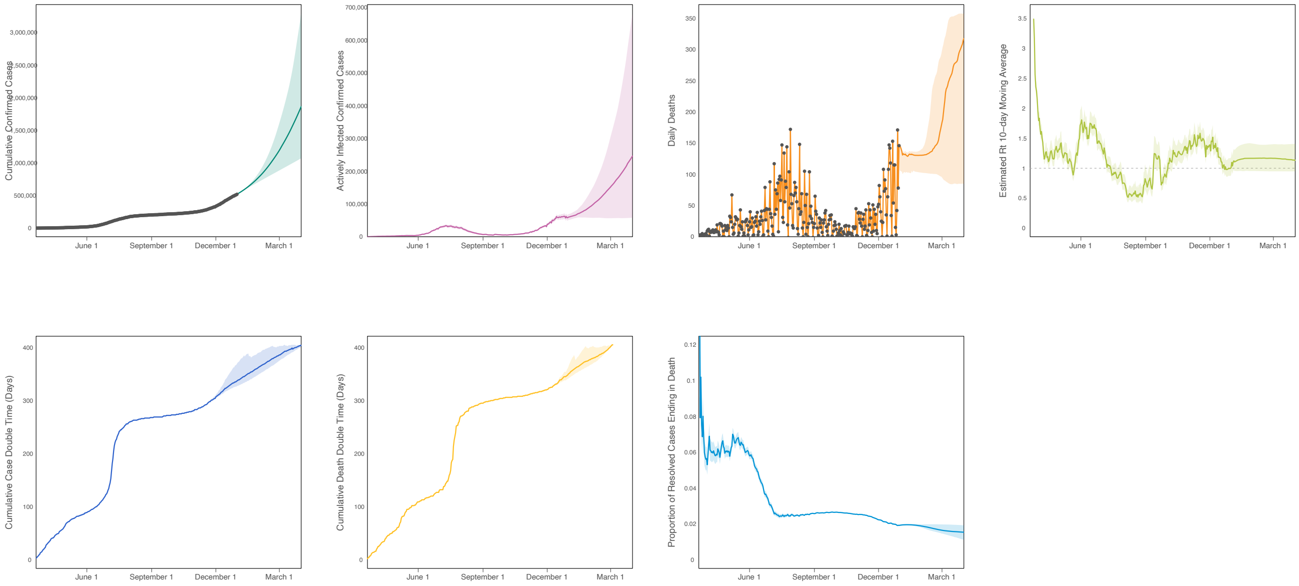

Cumulative Confirmed Cases
Actively Infected Confirmed Cases
Daily Deaths
Estimated Rt 10-day Moving Average
Cumulative Case Double Time (Days)
Cumulative Death Double Time (Days)
Proportion of Resolved Cases Ending in Death
June 1
September 1
December 1
March 1

AR

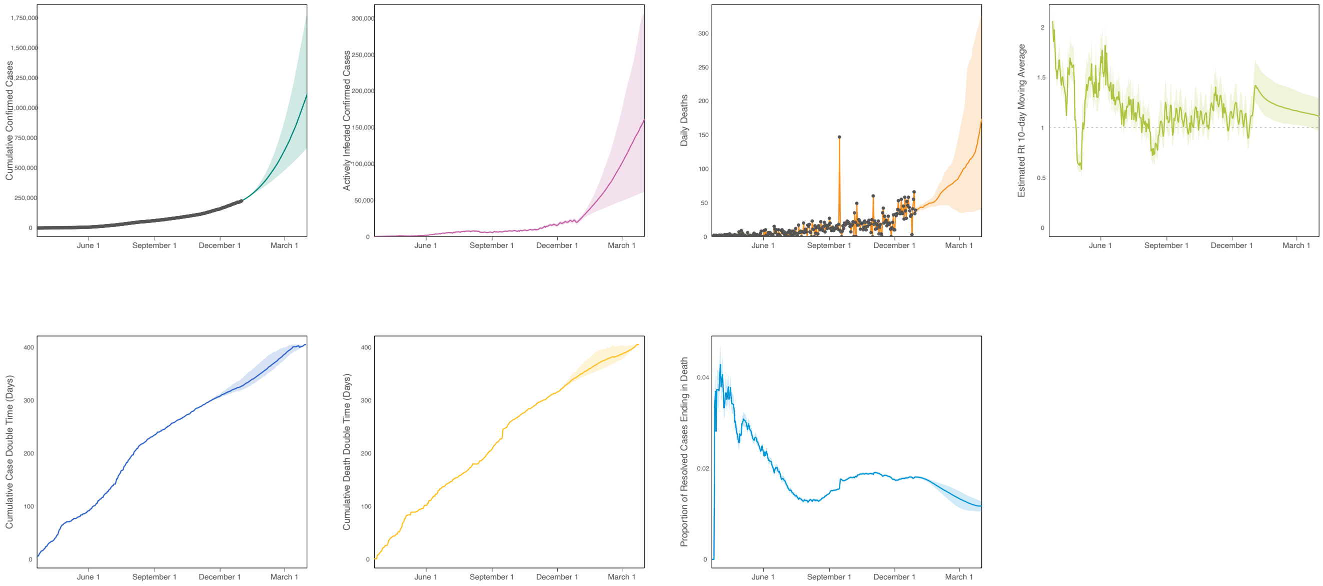

Cumulative Confirmed Cases
0
250,000
500,000
750,000
1,000,000
1,250,000
1,500,000
1,750,000
Actively Infected Confirmed Cases
0
50,000
100,000
150,000
200,000
250,000
300,000
Daily Deaths
0
50
100
150
200
250
300
Estimated Rt 10-day Moving Average
0
0.5
1
1.5
2
Cumulative Case Double Time (Days)
0
100
200
300
400
Cumulative Death Double Time (Days)
Proportion of Resolved Cases Ending in Death
0
0.02
0.04
June 1
September 1
December 1
March 1

CA

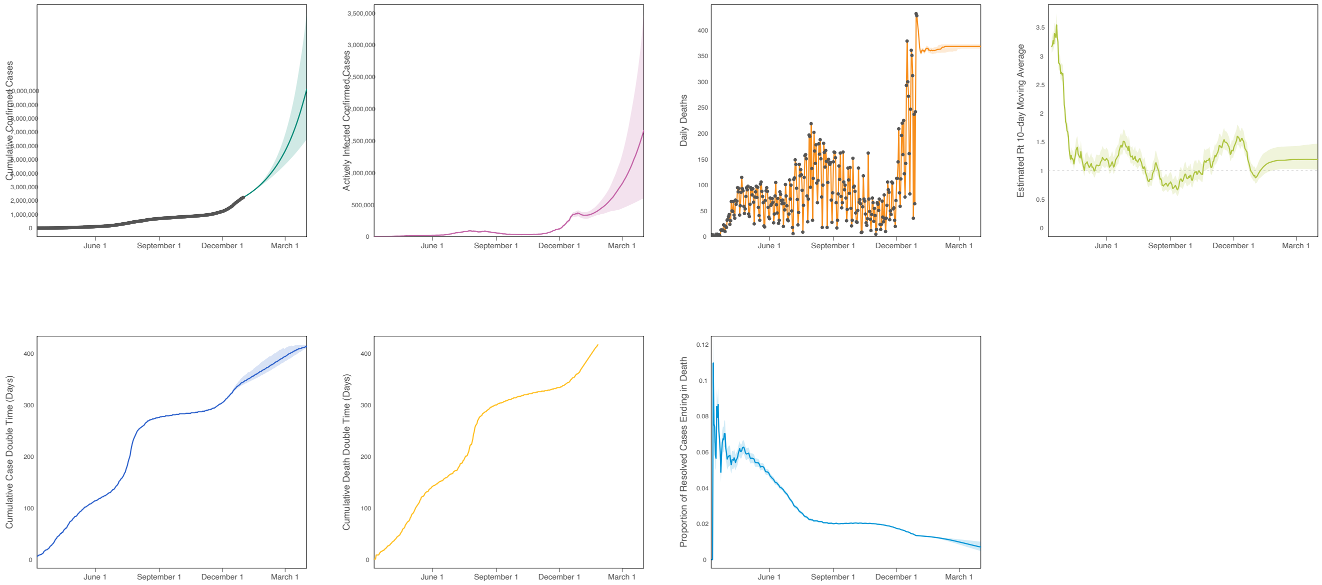

Cumulative Confirmed Cases
0
1,000,000
2,000,000
3,000,000
Actively Infected Confirmed Cases
0
500,000
1,000,000
1,500,000
2,000,000
2,500,000
3,000,000
3,500,000
Daily Deaths
0
50
100
150
200
250
300
350
400
Estimated Rt 10-day Moving Average
0
0.5
1
1.5
2
2.5
3
3.5
Cumulative Case Double Time (Days)
0
100
200
300
400
Cumulative Death Double Time (Days)
Proportion of Resolved Cases Ending in Death
0
0.02
0.04
0.06
0.08
0.1
0.12
June 1
September 1
December 1
March 1

CO

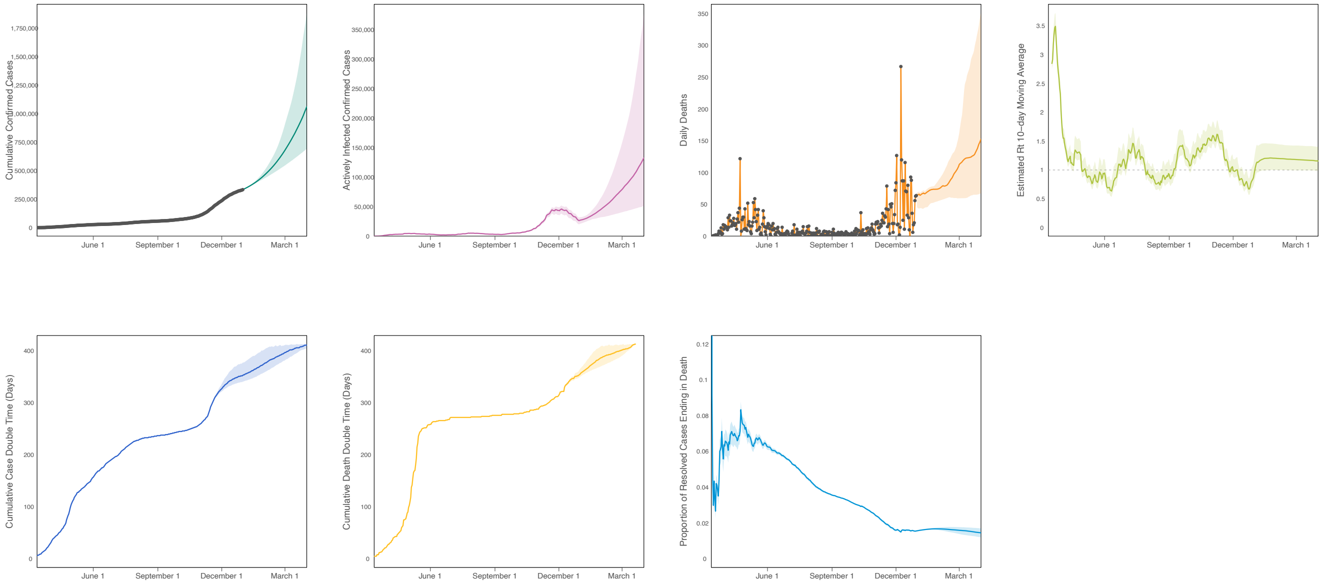

Cumulative Confirmed Cases
0
250,000
500,000
750,000
1,000,000
1,250,000
1,500,000
1,750,000
Actively Infected Confirmed Cases
0
50,000
100,000
150,000
200,000
250,000
300,000
350,000
Daily Deaths
0
50
100
150
200
250
300
350
Estimated Rt 10-day Moving Average
0
0.5
1
1.5
2
2.5
3
3.5
Cumulative Case Double Time (Days)
0
100
200
300
400
Cumulative Death Double Time (Days)
Proportion of Resolved Cases Ending in Death
0
0.02
0.04
0.06
0.08
0.1
0.12
June 1
September 1
December 1
March 1

CT

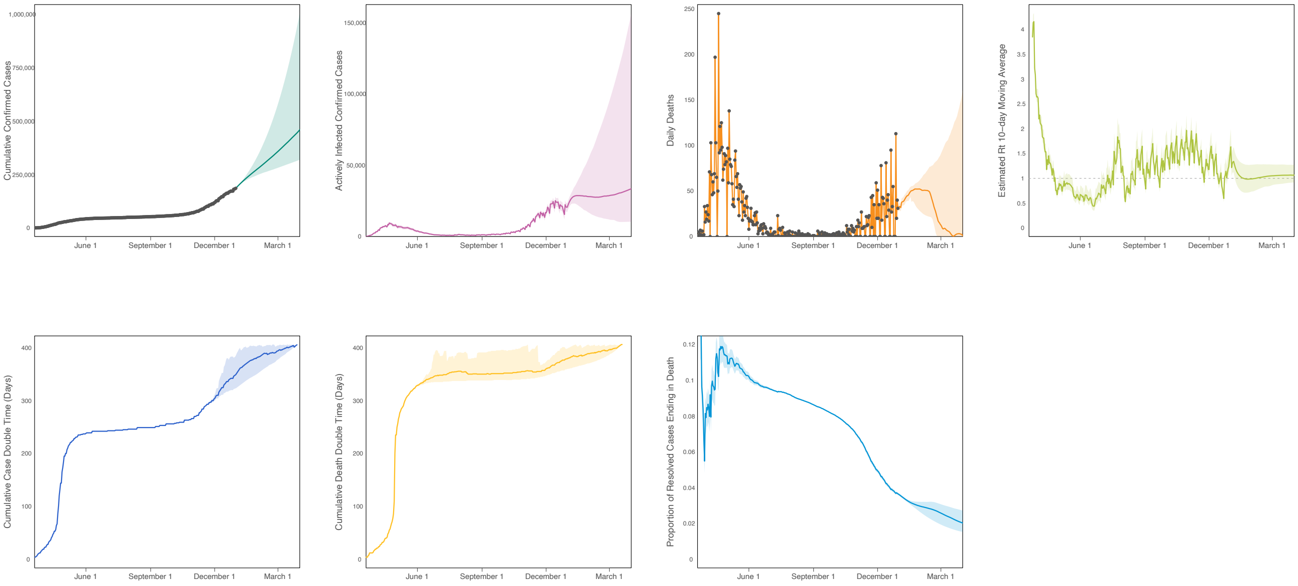
Cumulative Confirmed Cases
Actively Infected Confirmed Cases
Daily Deaths
Estimated Rt 10–day Moving Average
Cumulative Case Double Time (Days)
Cumulative Death Double Time (Days)
Proportion of Resolved Cases Ending in Death
June 1
September 1
December 1
March 1

DE

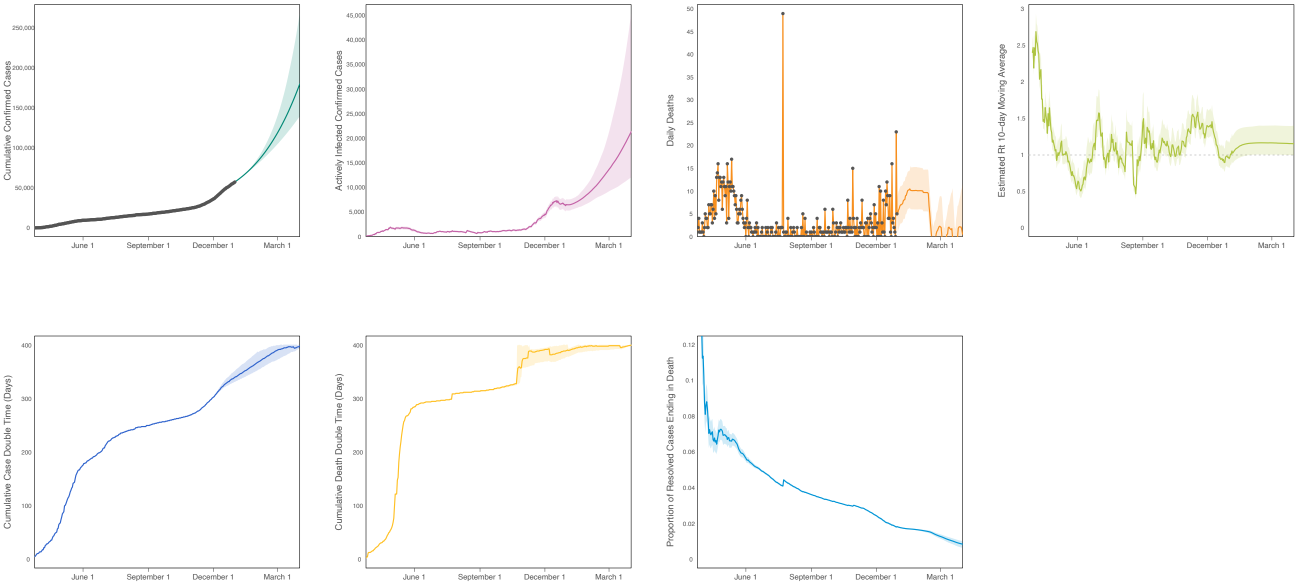
Cumulative Confirmed Cases
Actively Infected Confirmed Cases
Daily Deaths
Estimated Rt 10–day Moving Average
Cumulative Case Double Time (Days)
Cumulative Death Double Time (Days)
Proportion of Resolved Cases Ending in Death
June 1
September 1
December 1
March 1

FL

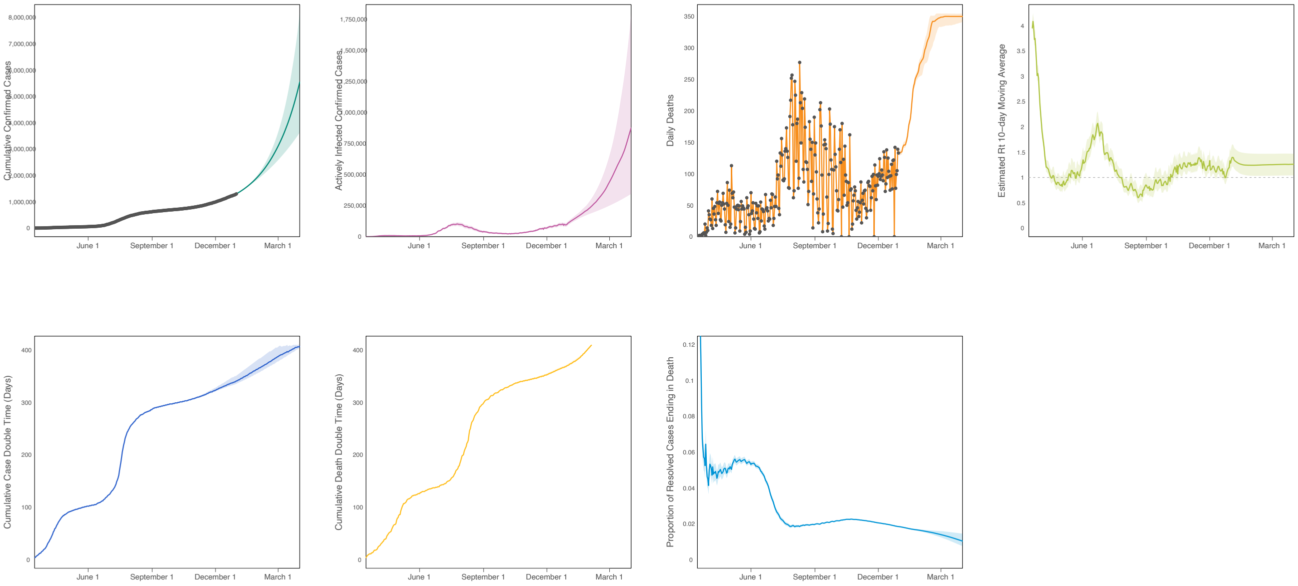
Cumulative Confirmed Cases
Actively Infected Confirmed Cases
Daily Deaths
Estimated Rt 10–day Moving Average
Cumulative Case Double Time (Days)
Cumulative Death Double Time (Days)
Proportion of Resolved Cases Ending in Death
June 1
September 1
December 1
March 1

GA

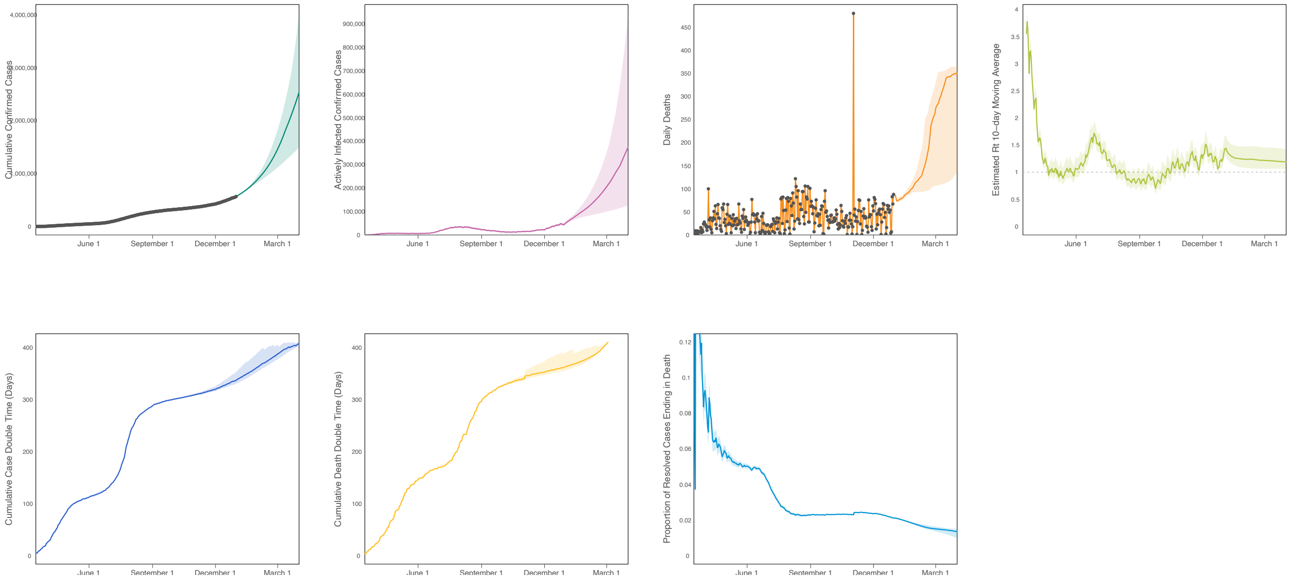
Cumulative Confirmed Cases
Actively Infected Confirmed Cases
Daily Deaths
Estimated Rt 10-day Moving Average
Cumulative Case Double Time (Days)
Cumulative Death Double Time (Days)
Proportion of Resolved Cases Ending in Death
June 1
September 1
December 1
March 1

HI

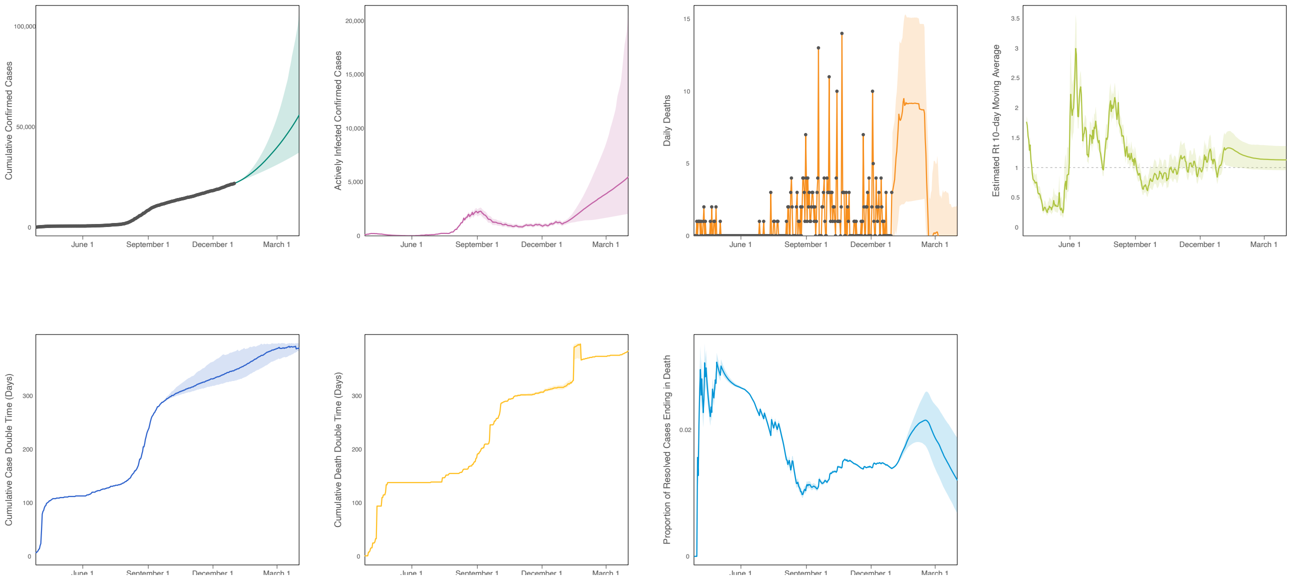
Cumulative Confirmed Cases
Actively Infected Confirmed Cases
Daily Deaths
Estimated Rt 10-day Moving Average
Cumulative Case Double Time (Days)
Cumulative Death Double Time (Days)
Proportion of Resolved Cases Ending in Death
June 1
September 1
December 1
March 1

ID

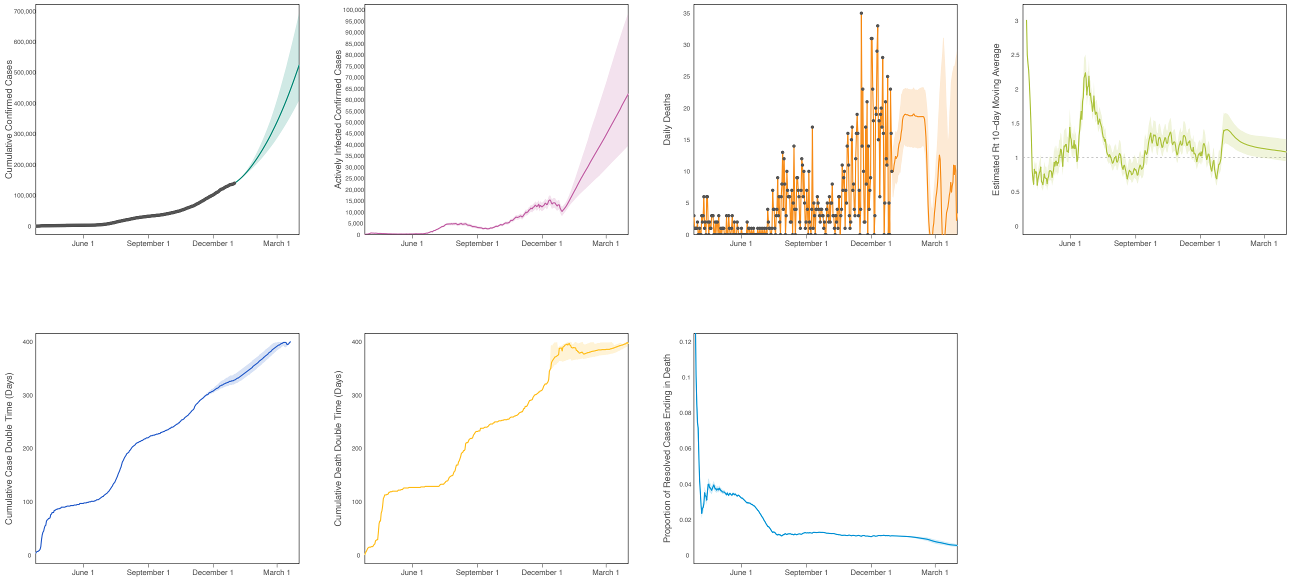
Cumulative Confirmed Cases
Actively Infected Confirmed Cases
Daily Deaths
Estimated Rt 10-day Moving Average
Cumulative Case Double Time (Days)
Cumulative Death Double Time (Days)
Proportion of Resolved Cases Ending in Death
June 1
September 1
December 1
March 1

IL

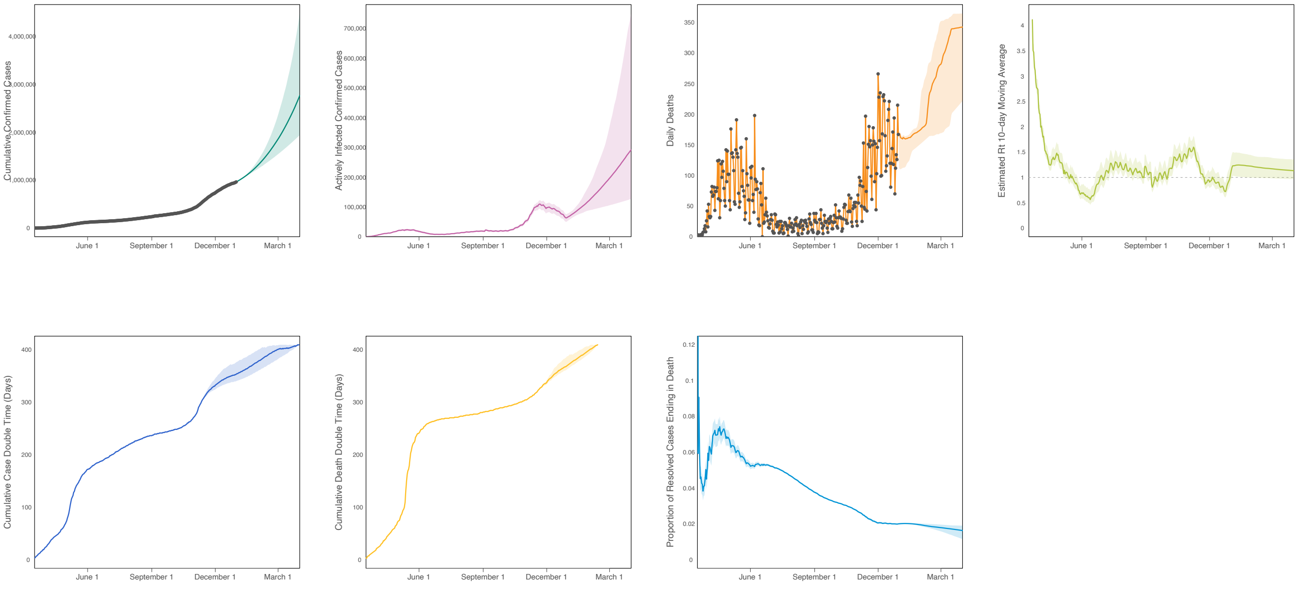

Cumulative Confirmed Cases
Actively Infected Confirmed Cases
Daily Deaths
Estimated Rt 10–day Moving Average
Cumulative Case Double Time (Days)
Cumulative Death Double Time (Days)
Proportion of Resolved Cases Ending in Death
June 1
September 1
December 1
March 1

IN

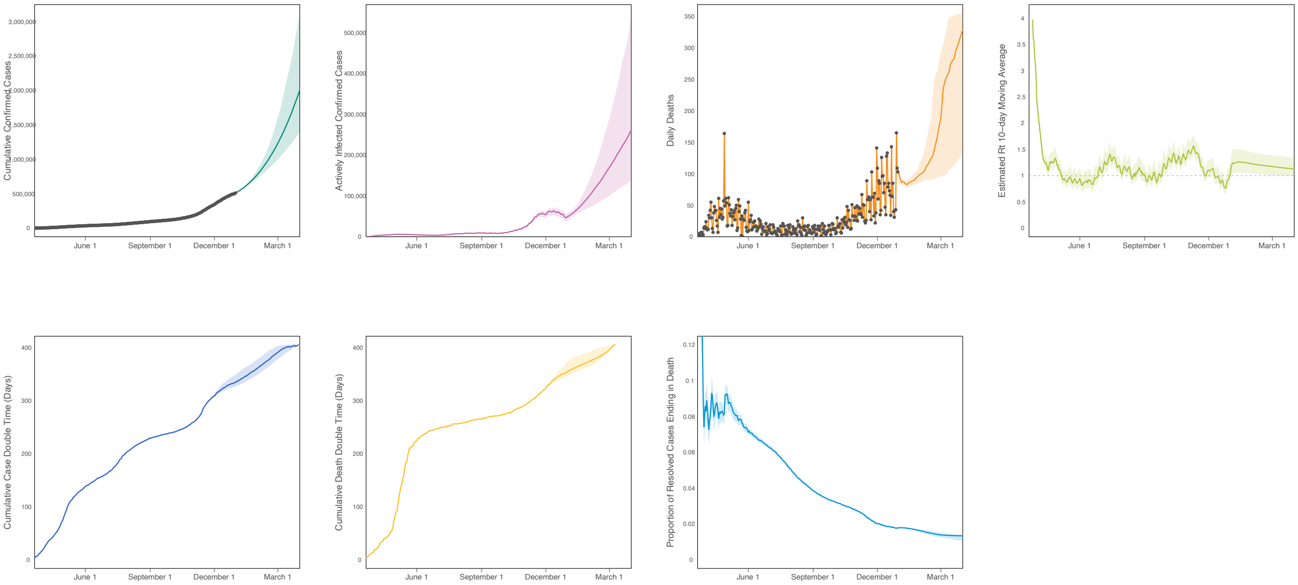

Cumulative Confirmed Cases
Actively Infected Confirmed Cases
Daily Deaths
Estimated Rt 10–day Moving Average
Cumulative Case Double Time (Days)
Cumulative Death Double Time (Days)
Proportion of Resolved Cases Ending in Death
June 1
September 1
December 1
March 1

IA

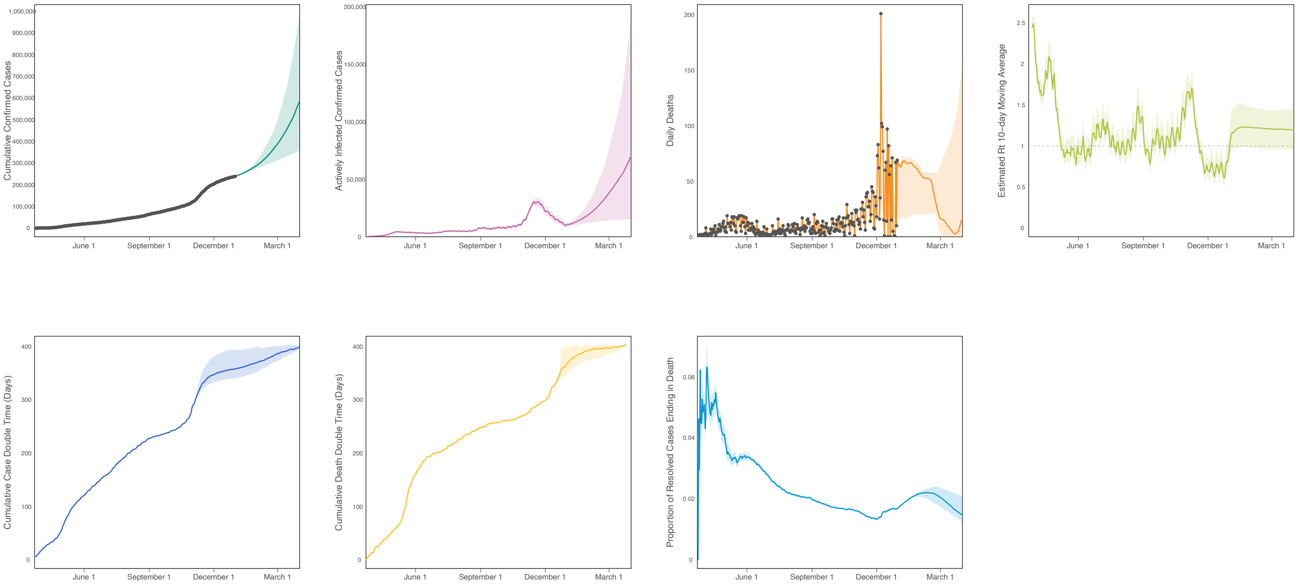

Cumulative Confirmed Cases
Actively Infected Confirmed Cases
Daily Deaths
Estimated Rt 10–day Moving Average
Cumulative Case Double Time (Days)
Cumulative Death Double Time (Days)
Proportion of Resolved Cases Ending in Death
June 1
September 1
December 1
March 1

KS

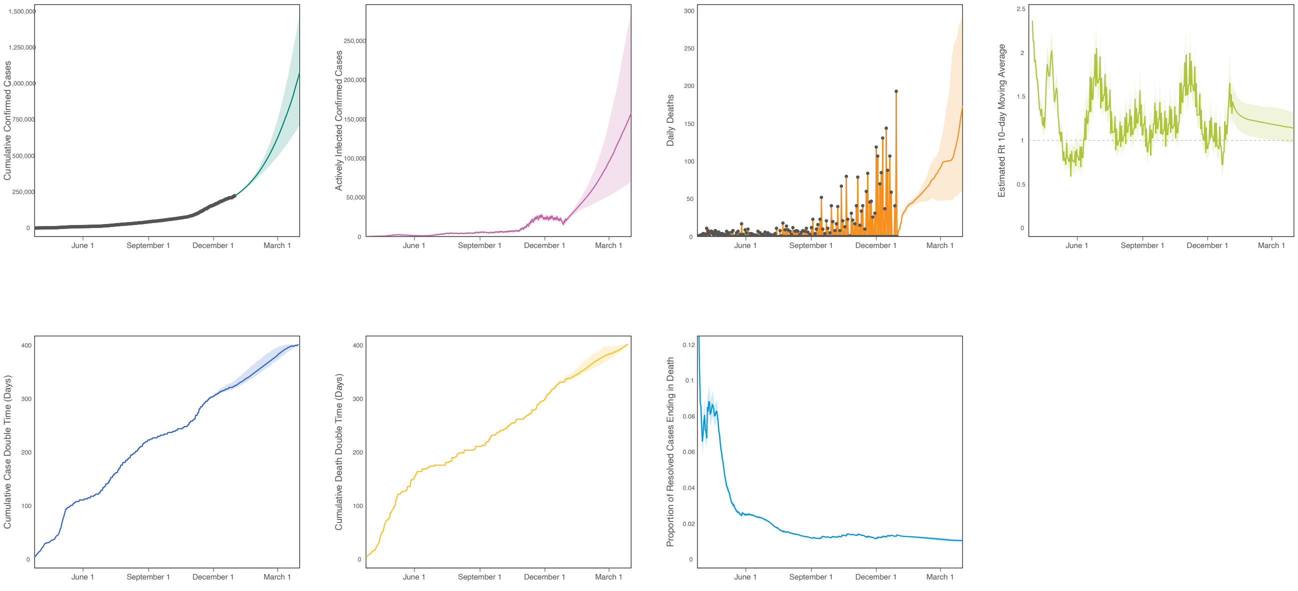
Cumulative Confirmed Cases
Actively Infected Confirmed Cases
Daily Deaths
Estimated Rt 10-day Moving Average
Cumulative Case Double Time (Days)
Cumulative Death Double Time (Days)
Proportion of Resolved Cases Ending in Death
June 1
September 1
December 1
March 1

KY

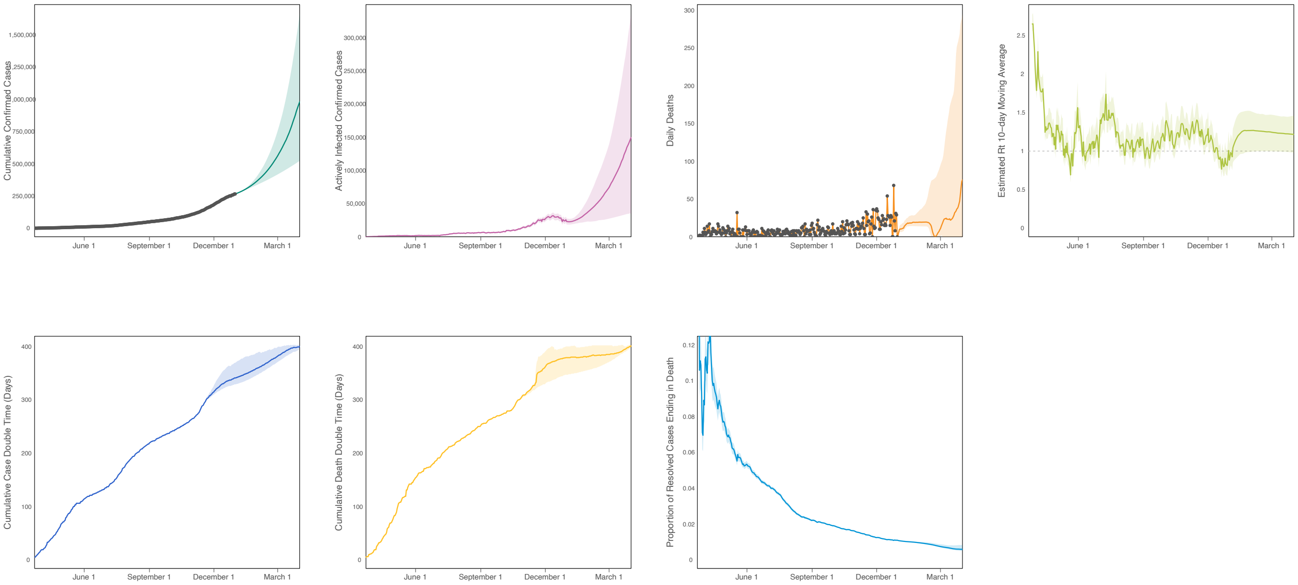
Cumulative Confirmed Cases
Actively Infected Confirmed Cases
Daily Deaths
Estimated Rt 10-day Moving Average
Cumulative Case Double Time (Days)
Cumulative Death Double Time (Days)
Proportion of Resolved Cases Ending in Death
June 1
September 1
December 1
March 1

LA

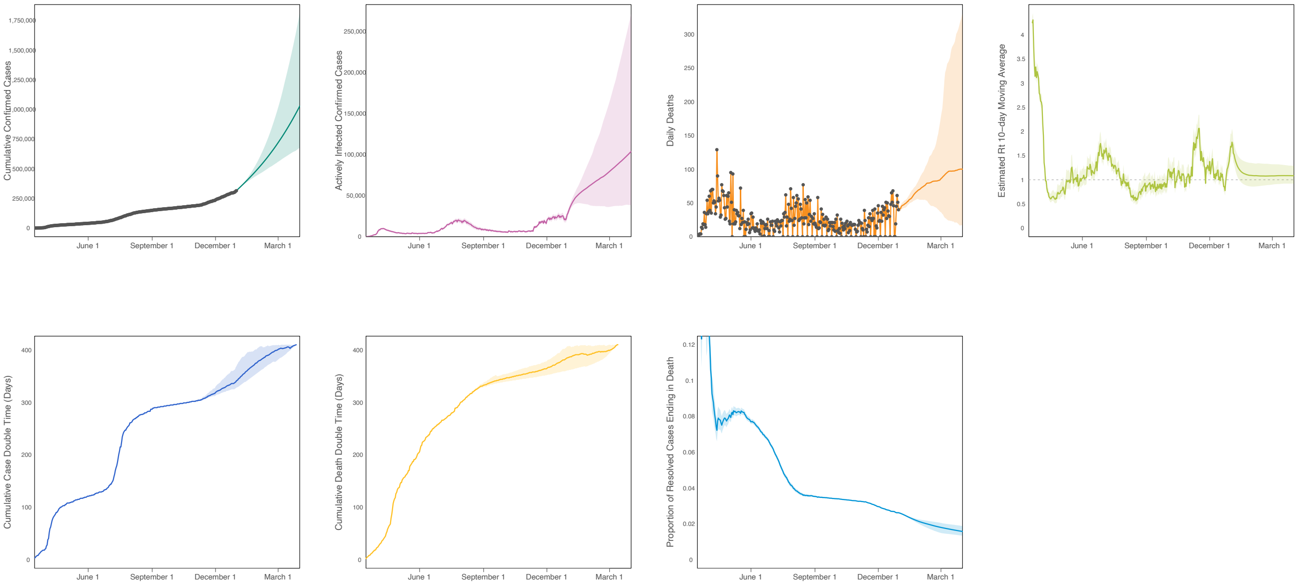
Cumulative Confirmed Cases
Actively Infected Confirmed Cases
Daily Deaths
Estimated Rt 10-day Moving Average
Cumulative Case Double Time (Days)
Cumulative Death Double Time (Days)
Proportion of Resolved Cases Ending in Death
June 1
September 1
December 1
March 1

ME

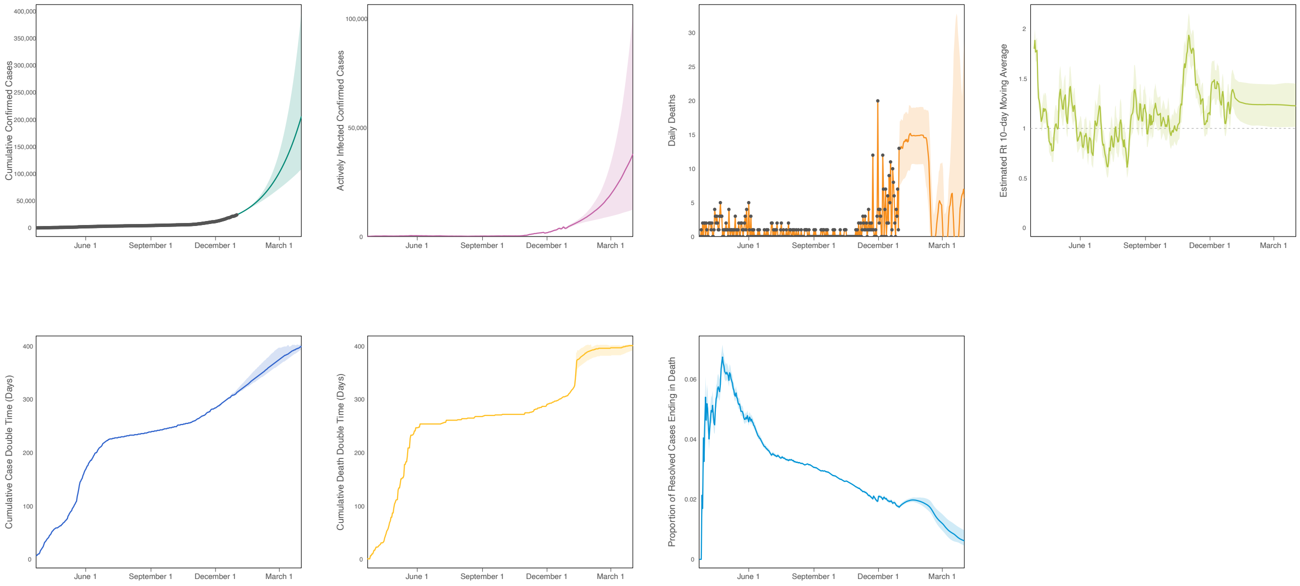
Cumulative Confirmed Cases
Actively Infected Confirmed Cases
Daily Deaths
Estimated Rt 10-day Moving Average
Cumulative Case Double Time (Days)
Cumulative Death Double Time (Days)
Proportion of Resolved Cases Ending in Death
June 1
September 1
December 1
March 1

MD

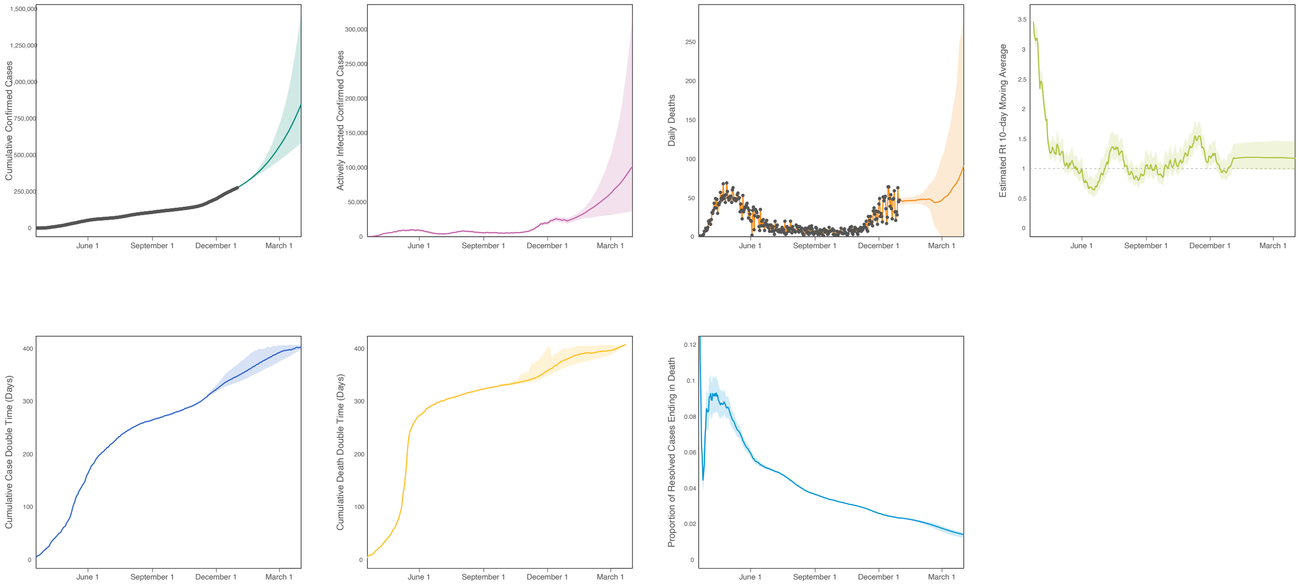
Cumulative Confirmed Cases
Actively Infected Confirmed Cases
Daily Deaths
Estimated Rt 10-day Moving Average
Cumulative Case Double Time (Days)
Cumulative Death Double Time (Days)
Proportion of Resolved Cases Ending in Death
June 1
September 1
December 1
March 1

MA

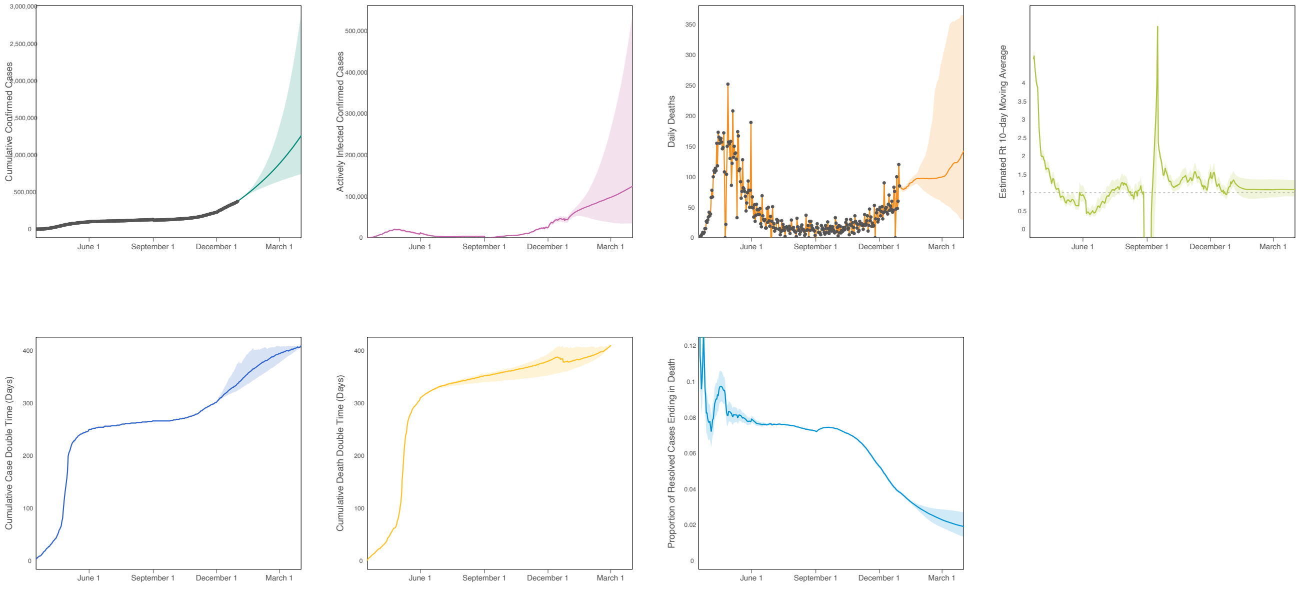
Cumulative Confirmed Cases
Actively Infected Confirmed Cases
Daily Deaths
Estimated Rt 10-day Moving Average
Cumulative Case Double Time (Days)
Cumulative Death Double Time (Days)
Proportion of Resolved Cases Ending in Death
June 1
September 1
December 1
March 1

MI

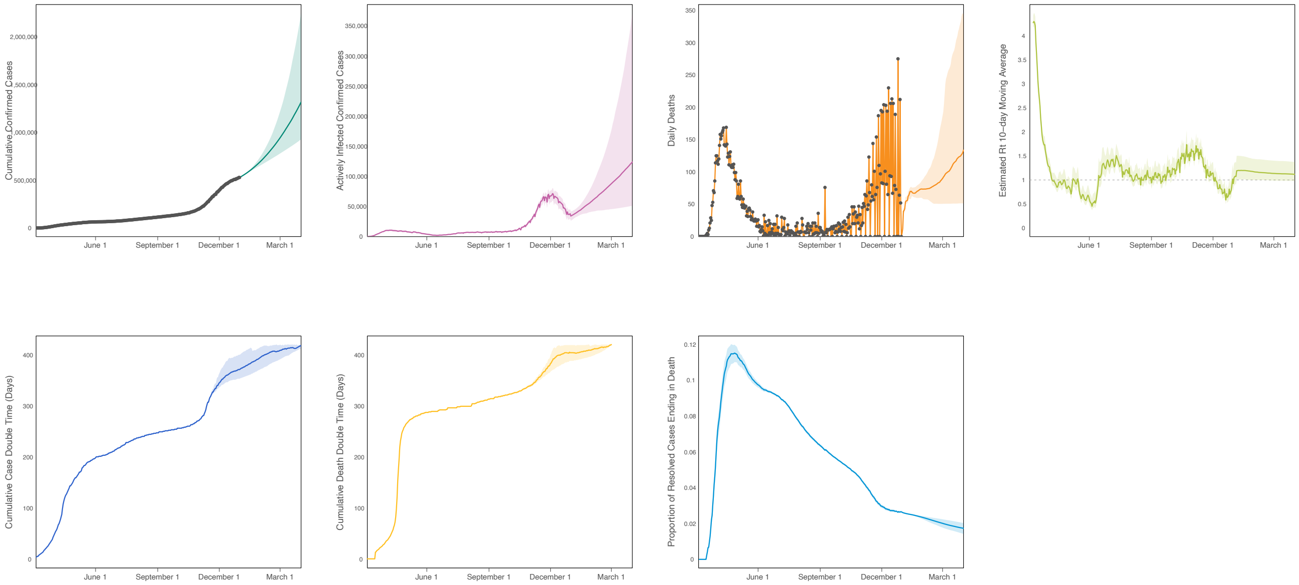
Cumulative Confirmed Cases
Actively Infected Confirmed Cases
Daily Deaths
Estimated Rt 10-day Moving Average
Cumulative Case Double Time (Days)
Cumulative Death Double Time (Days)
Proportion of Resolved Cases Ending in Death
June 1
September 1
December 1
March 1

MN

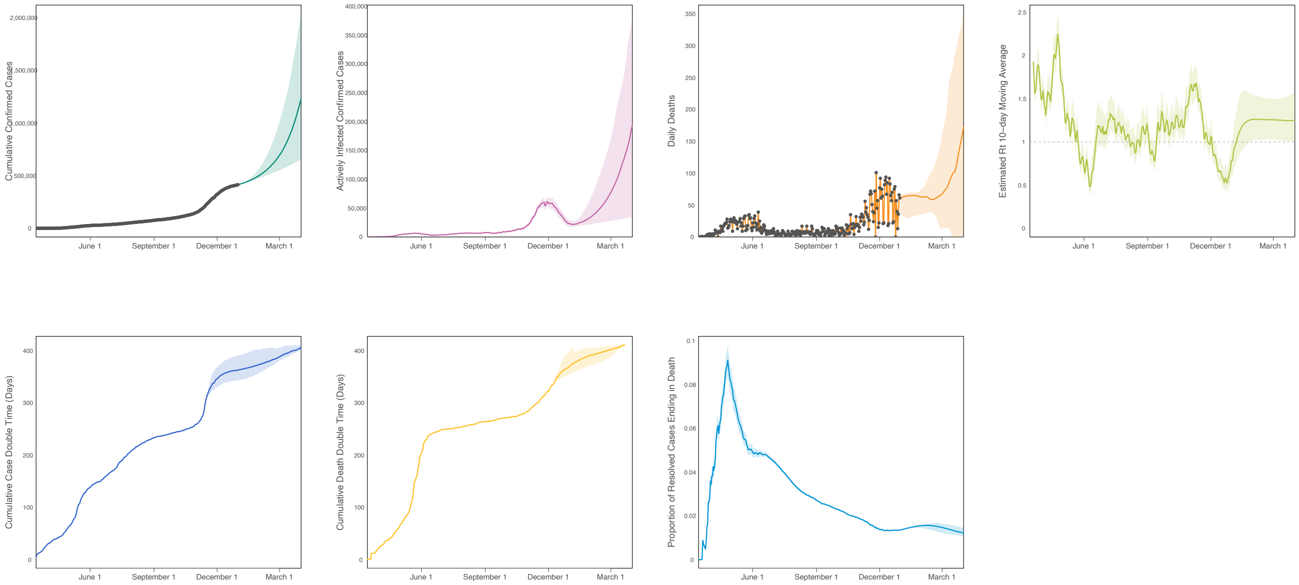
Cumulative Confirmed Cases
Actively Infected Confirmed Cases
Daily Deaths
Estimated Rt 10-day Moving Average
Cumulative Case Double Time (Days)
Cumulative Death Double Time (Days)
Proportion of Resolved Cases Ending in Death
June 1
September 1
December 1
March 1

MS

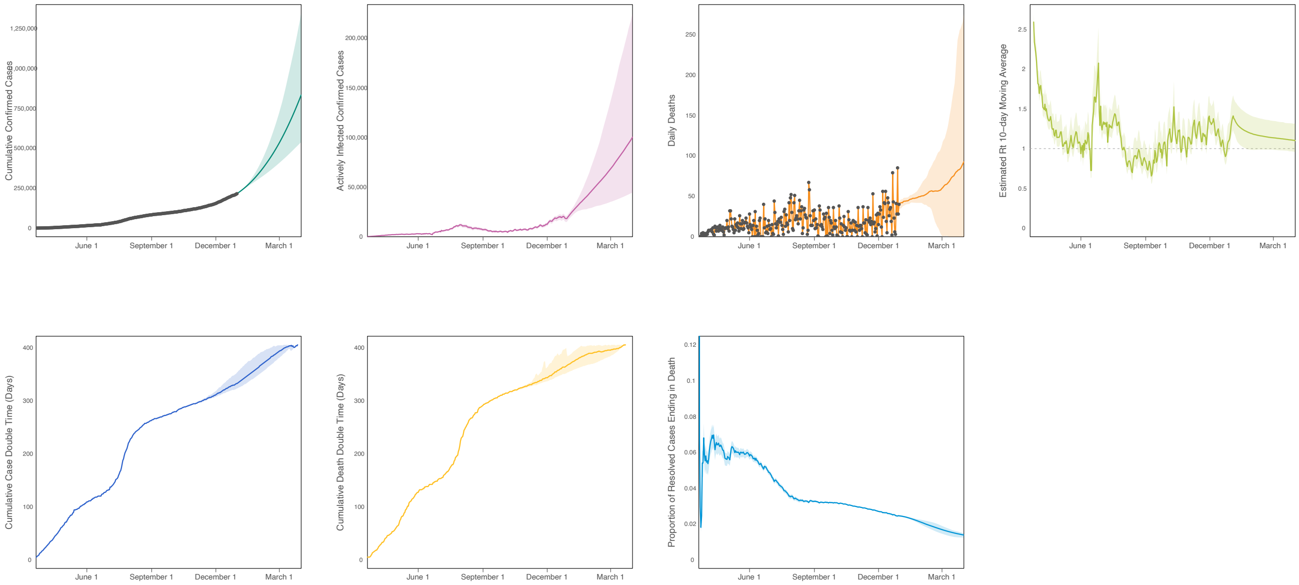
Cumulative Confirmed Cases
Actively Infected Confirmed Cases
Daily Deaths
Estimated Rt 10-day Moving Average
Cumulative Case Double Time (Days)
Cumulative Death Double Time (Days)
Proportion of Resolved Cases Ending in Death
June 1
September 1
December 1
March 1

MO

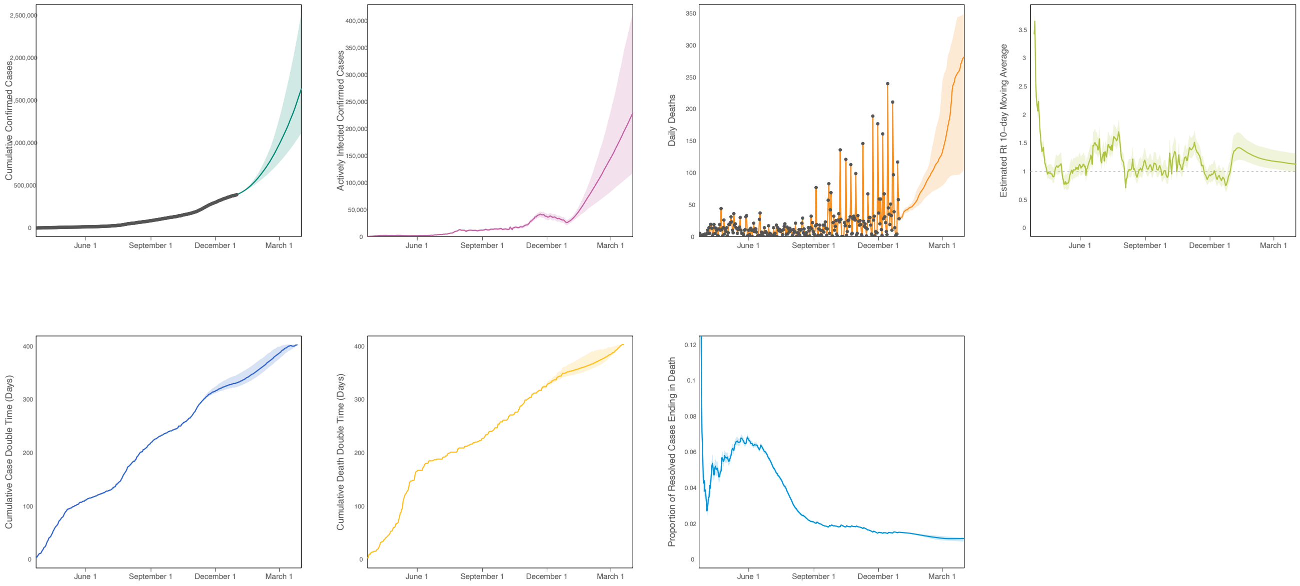
Cumulative Confirmed Cases
Actively Infected Confirmed Cases
Daily Deaths
Estimated Rt 10–day Moving Average
Cumulative Case Double Time (Days)
Cumulative Death Double Time (Days)
Proportion of Resolved Cases Ending in Death
June 1
September 1
December 1
March 1

MT

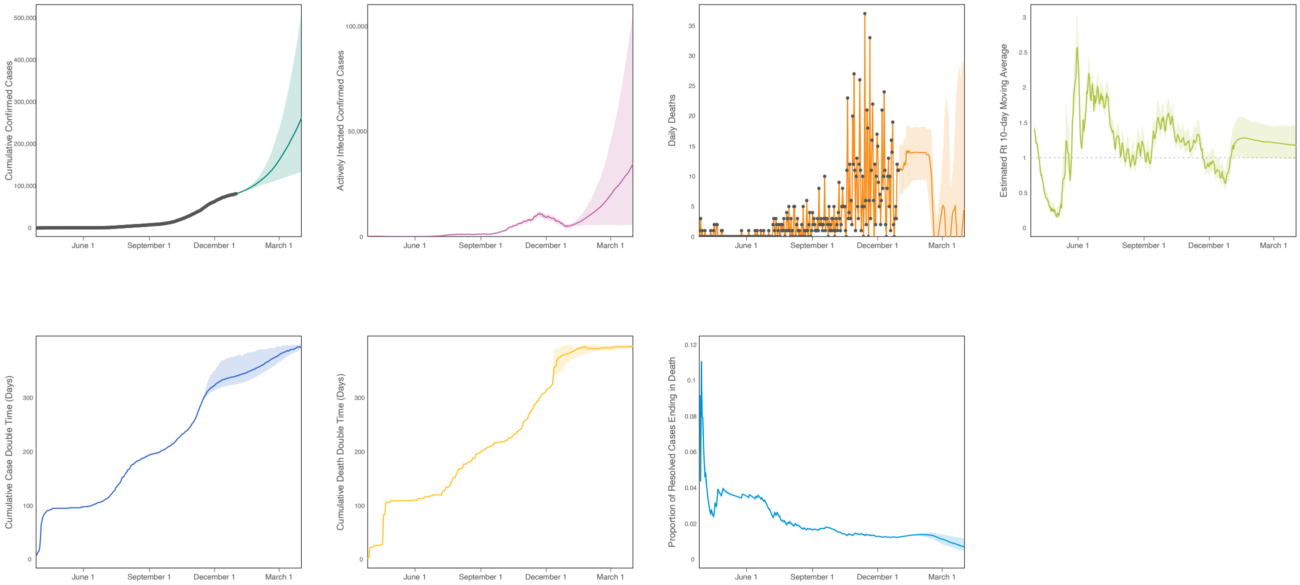
Cumulative Confirmed Cases
Actively Infected Confirmed Cases
Daily Deaths
Estimated Rt 10–day Moving Average
Cumulative Case Double Time (Days)
Cumulative Death Double Time (Days)
Proportion of Resolved Cases Ending in Death
June 1
September 1
December 1
March 1

NE

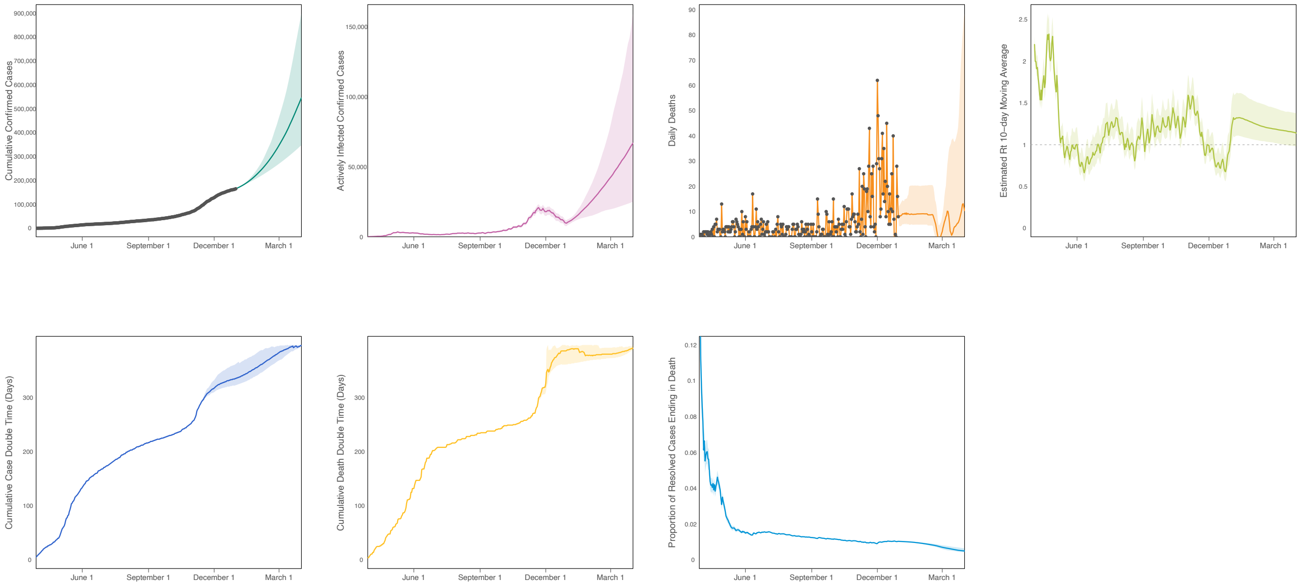
Cumulative Confirmed Cases
Actively Infected Confirmed Cases
Daily Deaths
Estimated Rt 10–day Moving Average
Cumulative Case Double Time (Days)
Cumulative Death Double Time (Days)
Proportion of Resolved Cases Ending in Death
June 1
September 1
December 1
March 1

NV

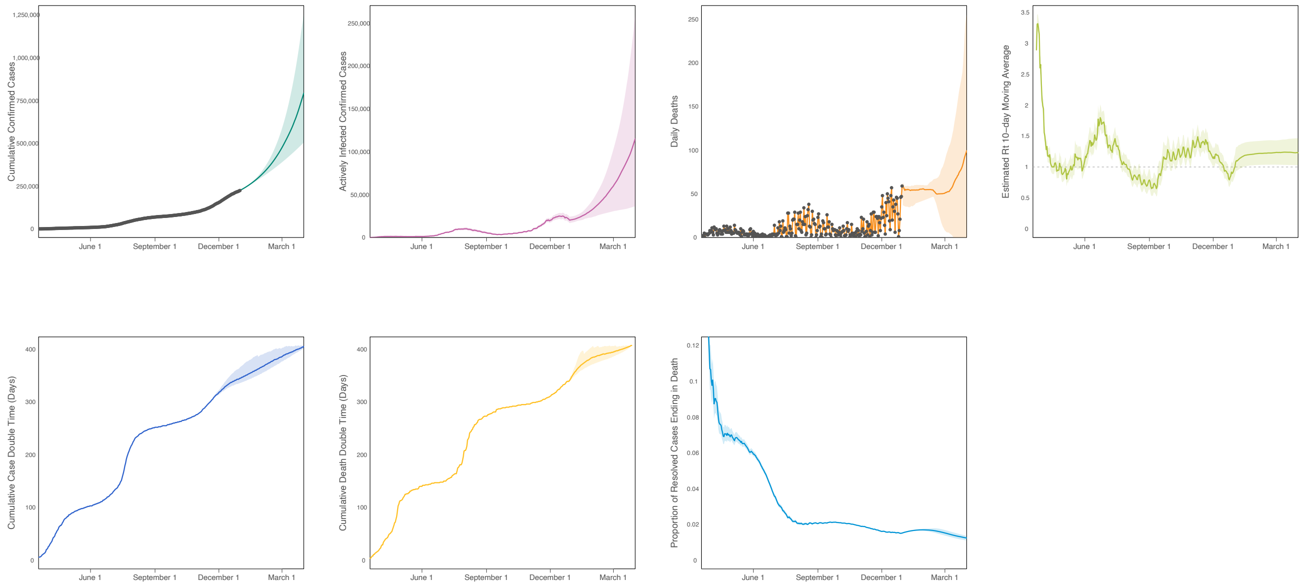

Cumulative Confirmed Cases
Actively Infected Confirmed Cases
Daily Deaths
Estimated Rt 10-day Moving Average
Cumulative Case Double Time (Days)
Cumulative Death Double Time (Days)
Proportion of Resolved Cases Ending in Death
June 1
September 1
December 1
March 1

NH

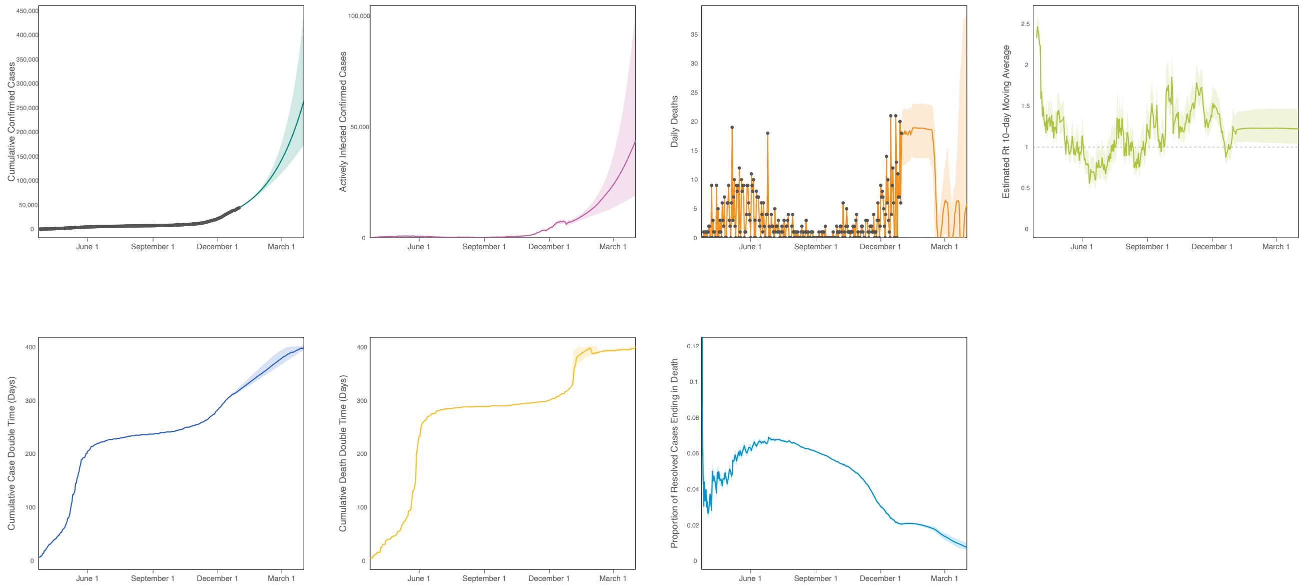

Cumulative Confirmed Cases
Actively Infected Confirmed Cases
Daily Deaths
Estimated Rt 10-day Moving Average
Cumulative Case Double Time (Days)
Cumulative Death Double Time (Days)
Proportion of Resolved Cases Ending in Death
June 1
September 1
December 1
March 1

NJ

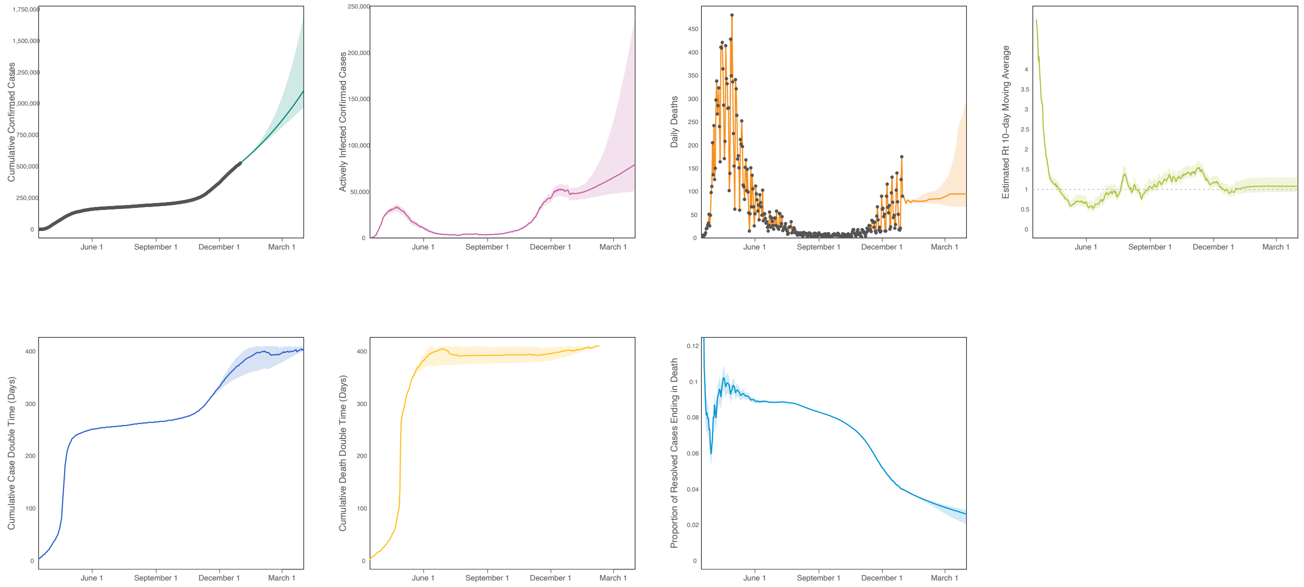

Cumulative Confirmed Cases
Actively Infected Confirmed Cases
Daily Deaths
Estimated Rt 10-day Moving Average
Cumulative Case Double Time (Days)
Cumulative Death Double Time (Days)
Proportion of Resolved Cases Ending in Death
June 1
September 1
December 1
March 1

NM

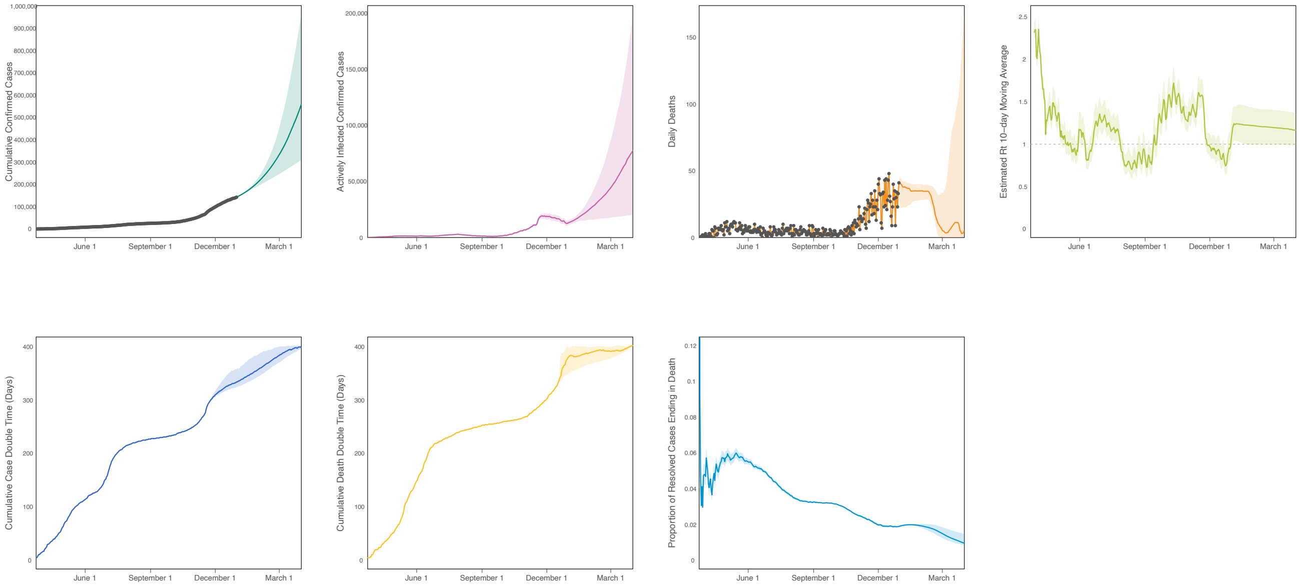
Cumulative Confirmed Cases
Actively Infected Confirmed Cases
Daily Deaths
Estimated Rt 10-day Moving Average
Cumulative Case Double Time (Days)
Cumulative Death Double Time (Days)
Proportion of Resolved Cases Ending in Death
June 1
September 1
December 1
March 1

NY

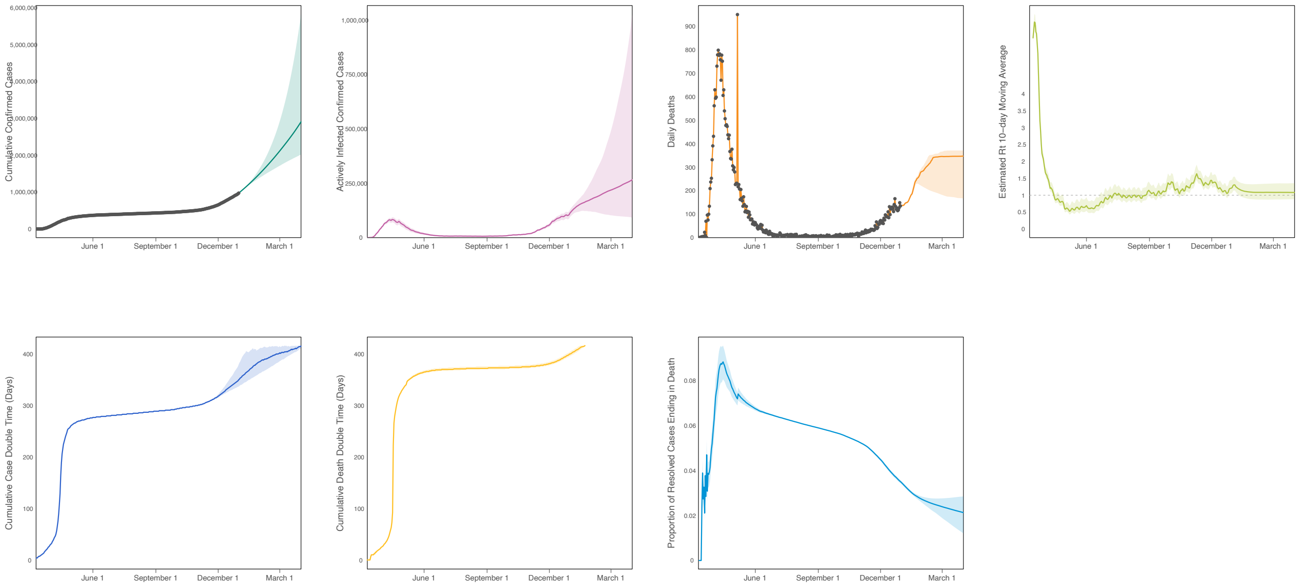
Cumulative Confirmed Cases
Actively Infected Confirmed Cases
Daily Deaths
Estimated Rt 10-day Moving Average
Cumulative Case Double Time (Days)
Cumulative Death Double Time (Days)
Proportion of Resolved Cases Ending in Death
June 1
September 1
December 1
March 1

NC

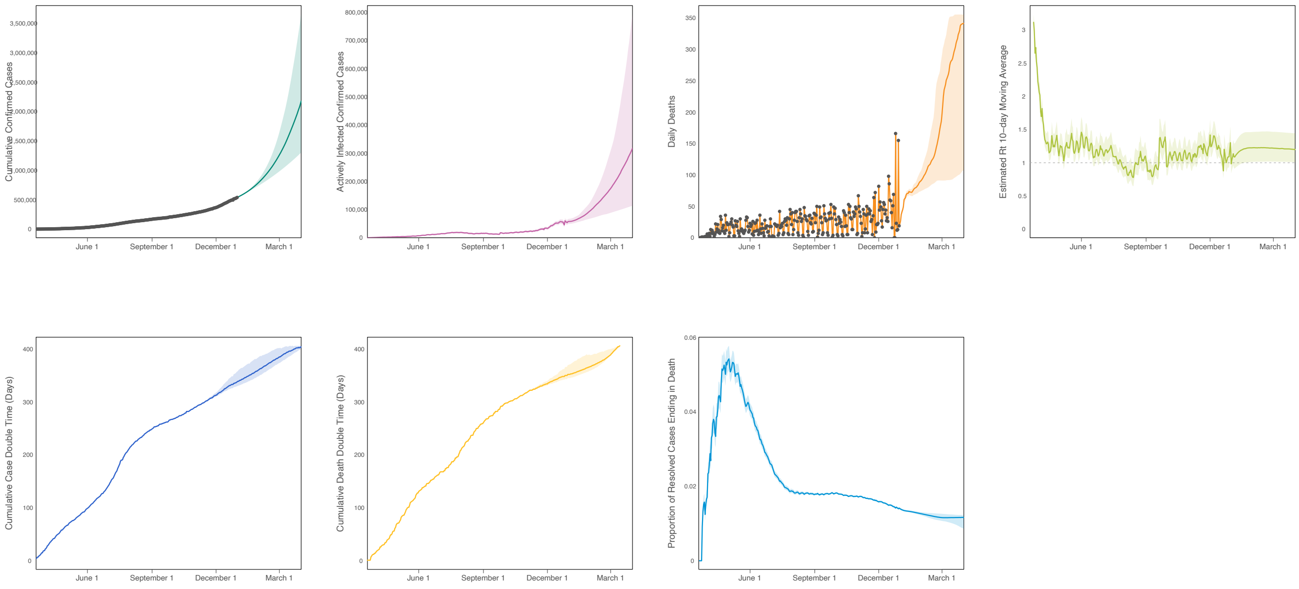
Cumulative Confirmed Cases
Actively Infected Confirmed Cases
Daily Deaths
Estimated Rt 10-day Moving Average
Cumulative Case Double Time (Days)
Cumulative Death Double Time (Days)
Proportion of Resolved Cases Ending in Death
June 1
September 1
December 1
March 1

ND

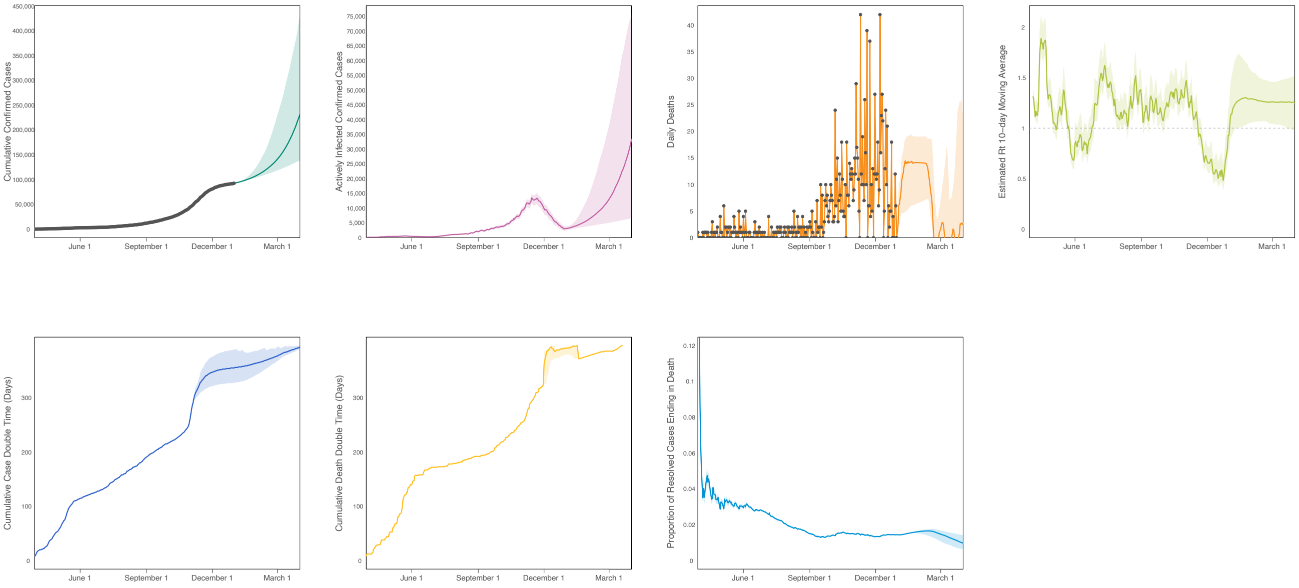

Cumulative Confirmed Cases
Actively Infected Confirmed Cases
Daily Deaths
Estimated Rt 10-day Moving Average
Cumulative Case Double Time (Days)
Cumulative Death Double Time (Days)
Proportion of Resolved Cases Ending in Death
June 1
September 1
December 1
March 1

OH

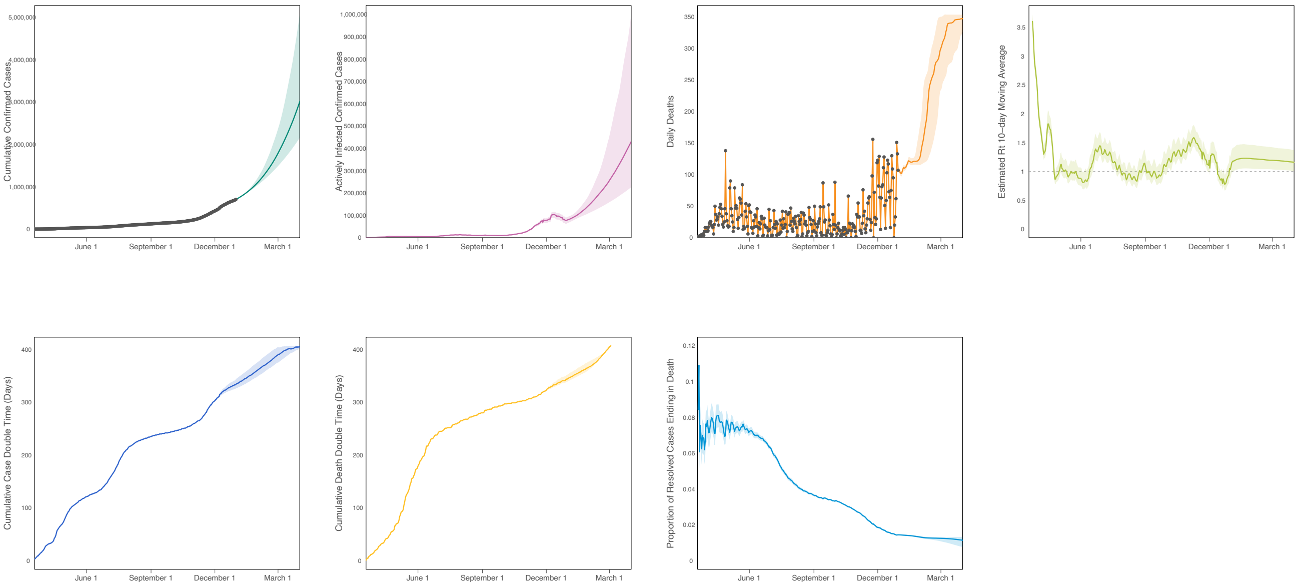

Cumulative Confirmed Cases
Actively Infected Confirmed Cases
Daily Deaths
Estimated Rt 10-day Moving Average
Cumulative Case Double Time (Days)
Cumulative Death Double Time (Days)
Proportion of Resolved Cases Ending in Death
June 1
September 1
December 1
March 1

OK

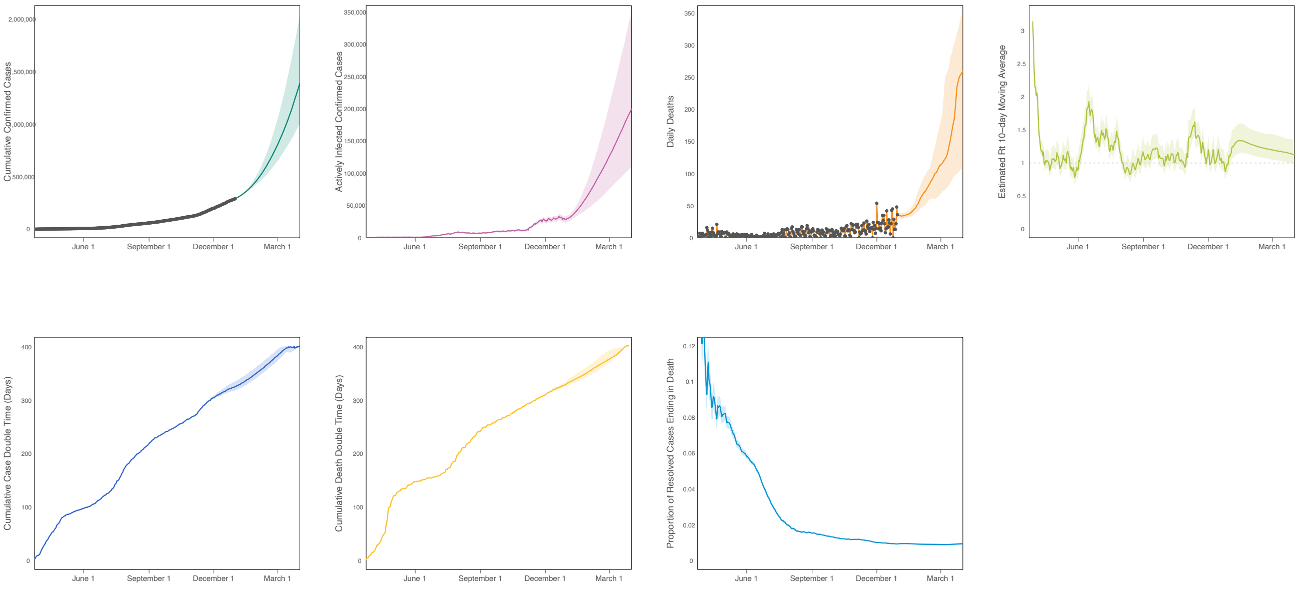

Cumulative Confirmed Cases
Actively Infected Confirmed Cases
Daily Deaths
Estimated Rt 10-day Moving Average
Cumulative Case Double Time (Days)
Cumulative Death Double Time (Days)
Proportion of Resolved Cases Ending in Death
June 1
September 1
December 1
March 1

OR

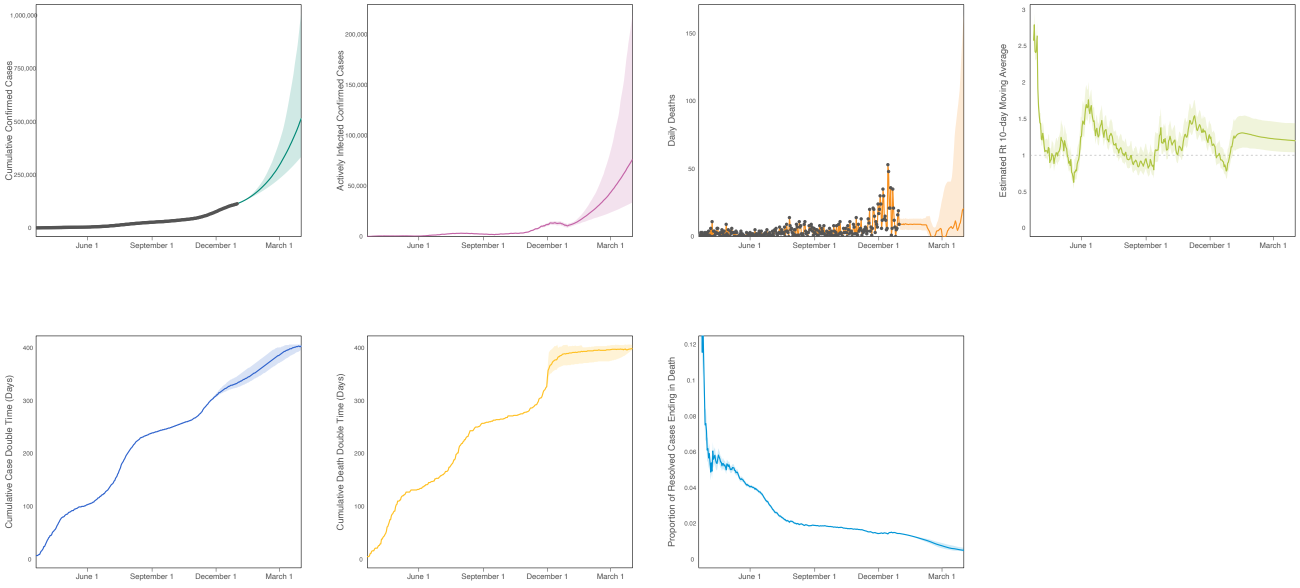
Cumulative Confirmed Cases
Actively Infected Confirmed Cases
Daily Deaths
Estimated Rt 10−day Moving Average
Cumulative Case Double Time (Days)
Cumulative Death Double Time (Days)
Proportion of Resolved Cases Ending in Death
June 1
September 1
December 1
March 1

PA

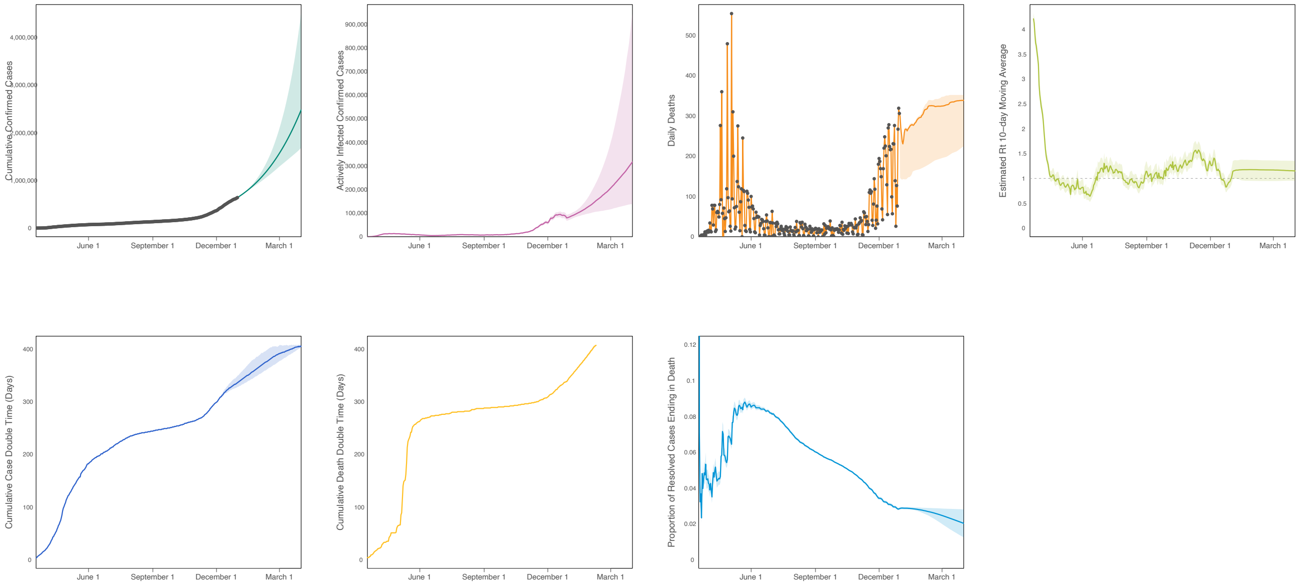
Cumulative Confirmed Cases
Actively Infected Confirmed Cases
Daily Deaths
Estimated Rt 10−day Moving Average
Cumulative Case Double Time (Days)
Cumulative Death Double Time (Days)
Proportion of Resolved Cases Ending in Death
June 1
September 1
December 1
March 1

RI

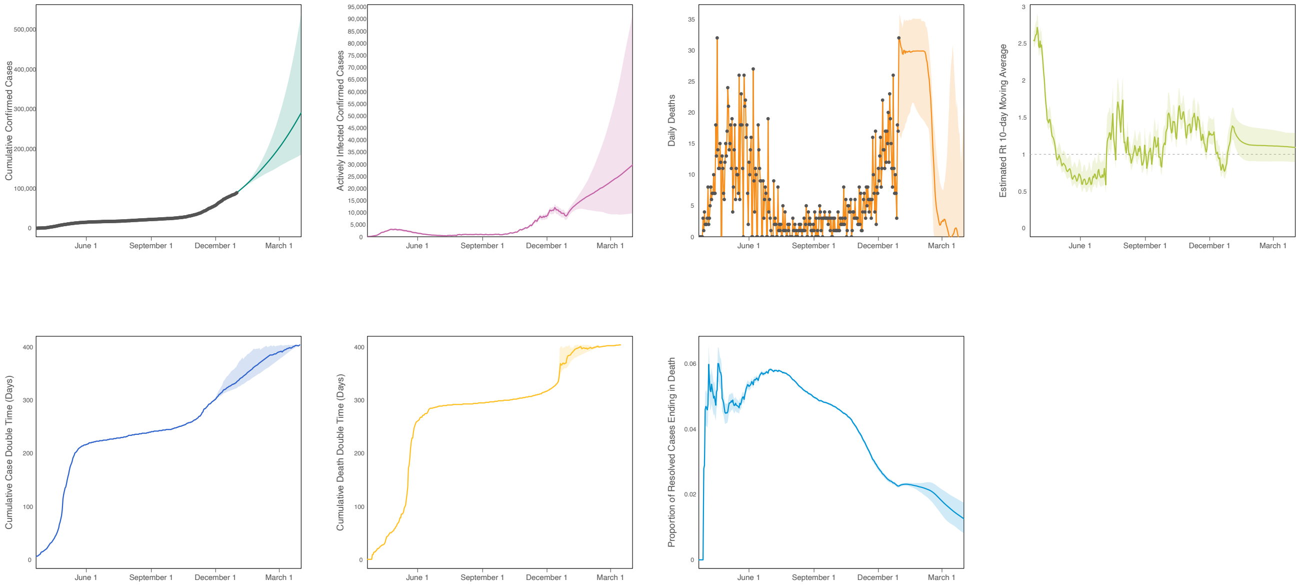
Cumulative Confirmed Cases
Actively Infected Confirmed Cases
Daily Deaths
Estimated Rt 10−day Moving Average
Cumulative Case Double Time (Days)
Cumulative Death Double Time (Days)
Proportion of Resolved Cases Ending in Death
June 1
September 1
December 1
March 1

SC

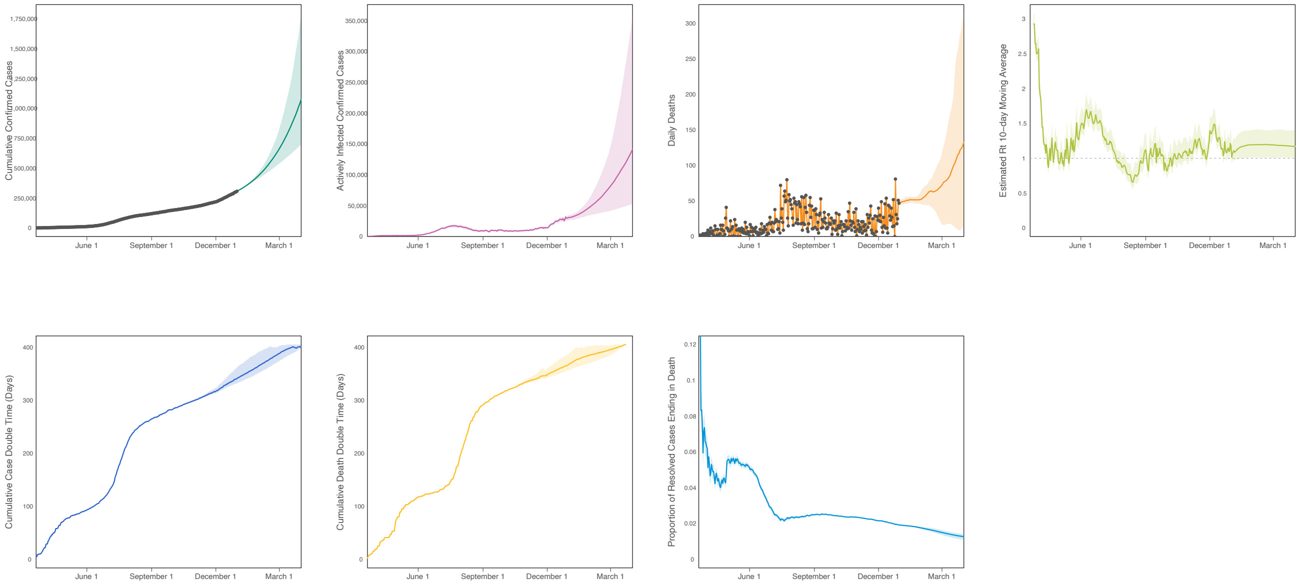

Cumulative Confirmed Cases
Actively Infected Confirmed Cases
Daily Deaths
Estimated Rt 10−day Moving Average
Cumulative Case Double Time (Days)
Cumulative Death Double Time (Days)
Proportion of Resolved Cases Ending in Death
June 1
September 1
December 1
March 1

SD

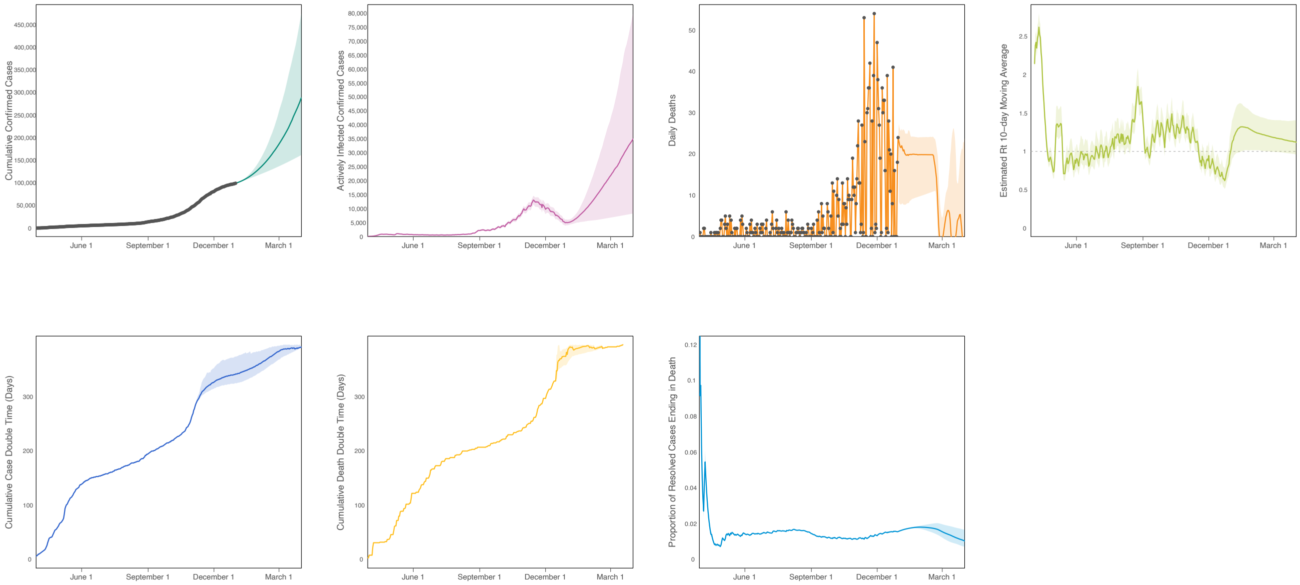

Cumulative Confirmed Cases
Actively Infected Confirmed Cases
Daily Deaths
Estimated Rt 10−day Moving Average
Cumulative Case Double Time (Days)
Cumulative Death Double Time (Days)
Proportion of Resolved Cases Ending in Death
June 1
September 1
December 1
March 1

TN

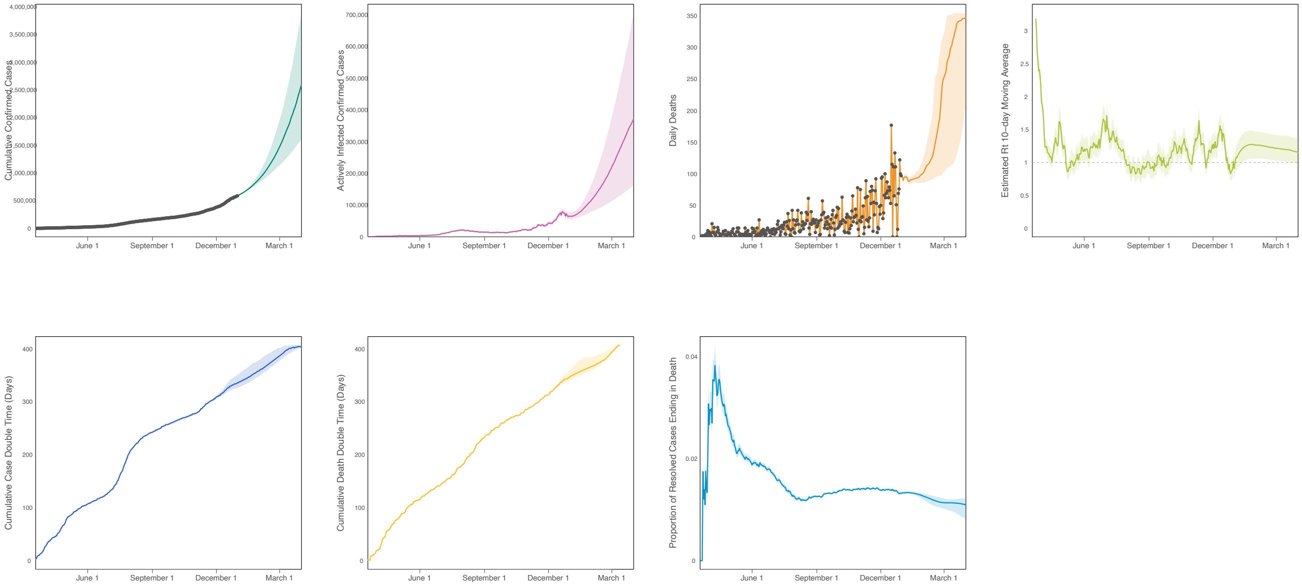

Cumulative Confirmed Cases
Actively Infected Confirmed Cases
Daily Deaths
Estimated Rt 10−day Moving Average
Cumulative Case Double Time (Days)
Cumulative Death Double Time (Days)
Proportion of Resolved Cases Ending in Death
June 1
September 1
December 1
March 1

TX

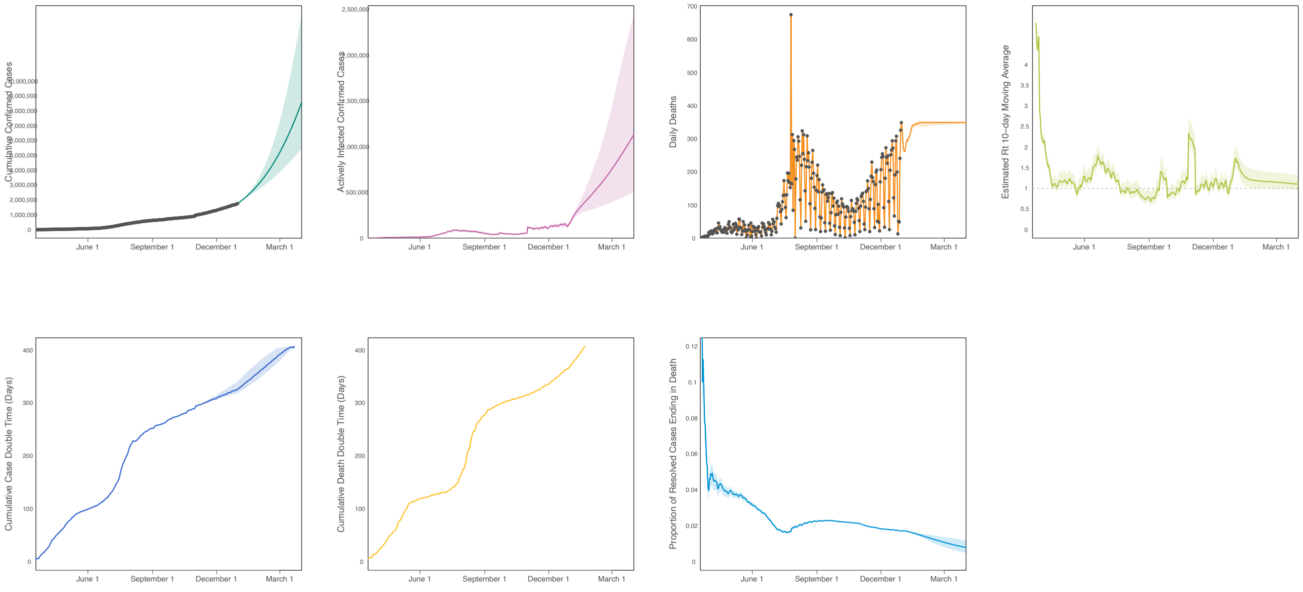
Cumulative Confirmed Cases
Actively Infected Confirmed Cases
Daily Deaths
Estimated Rt 10−day Moving Average
Cumulative Case Double Time (Days)
Cumulative Death Double Time (Days)
Proportion of Resolved Cases Ending in Death
June 1
September 1
December 1
March 1

UT

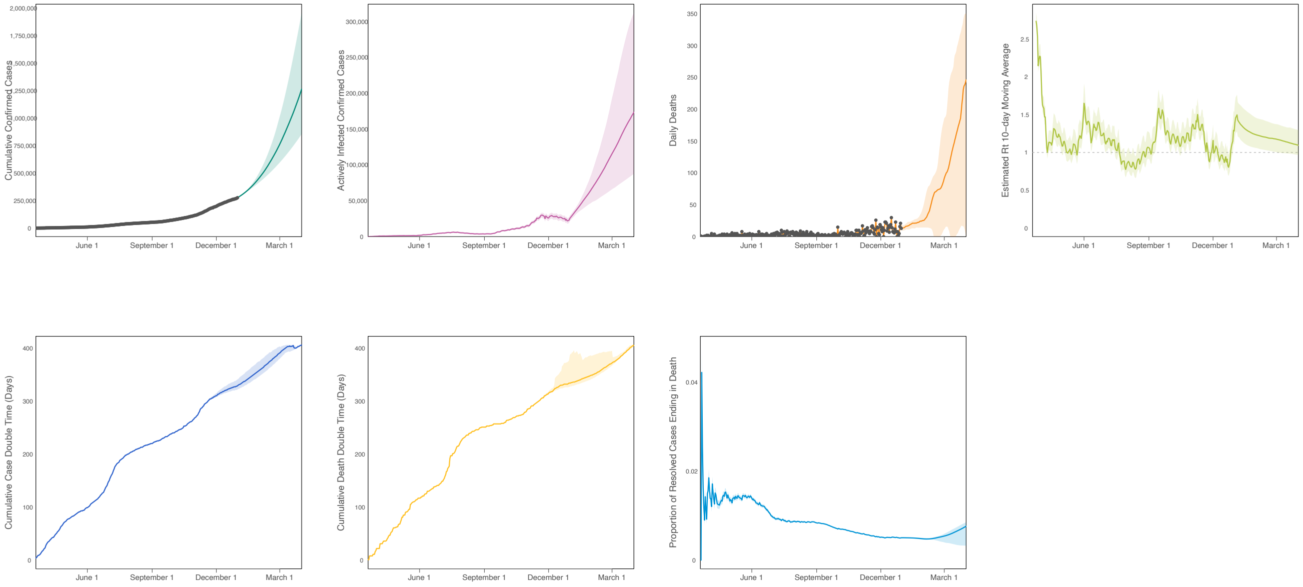
Cumulative Confirmed Cases
Actively Infected Confirmed Cases
Daily Deaths
Estimated Rt 10−day Moving Average
Cumulative Case Double Time (Days)
Cumulative Death Double Time (Days)
Proportion of Resolved Cases Ending in Death
June 1
September 1
December 1
March 1

VT

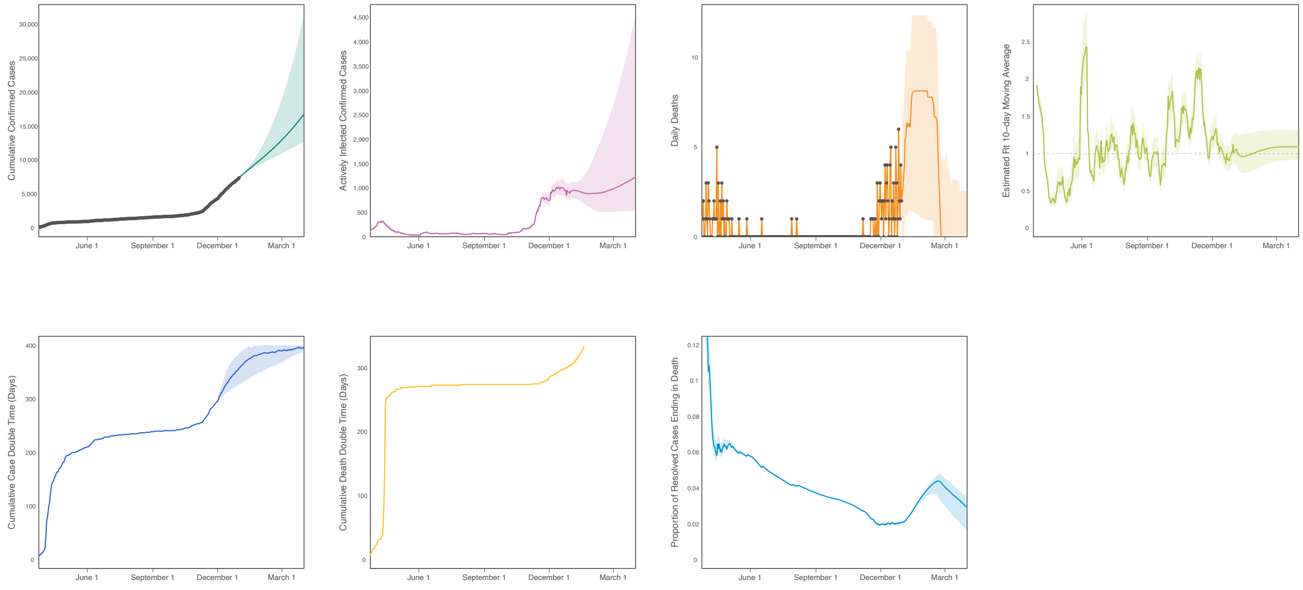
Cumulative Confirmed Cases
Actively Infected Confirmed Cases
Daily Deaths
Estimated Rt 10−day Moving Average
Cumulative Case Double Time (Days)
Cumulative Death Double Time (Days)
Proportion of Resolved Cases Ending in Death
June 1
September 1
December 1
March 1

VA

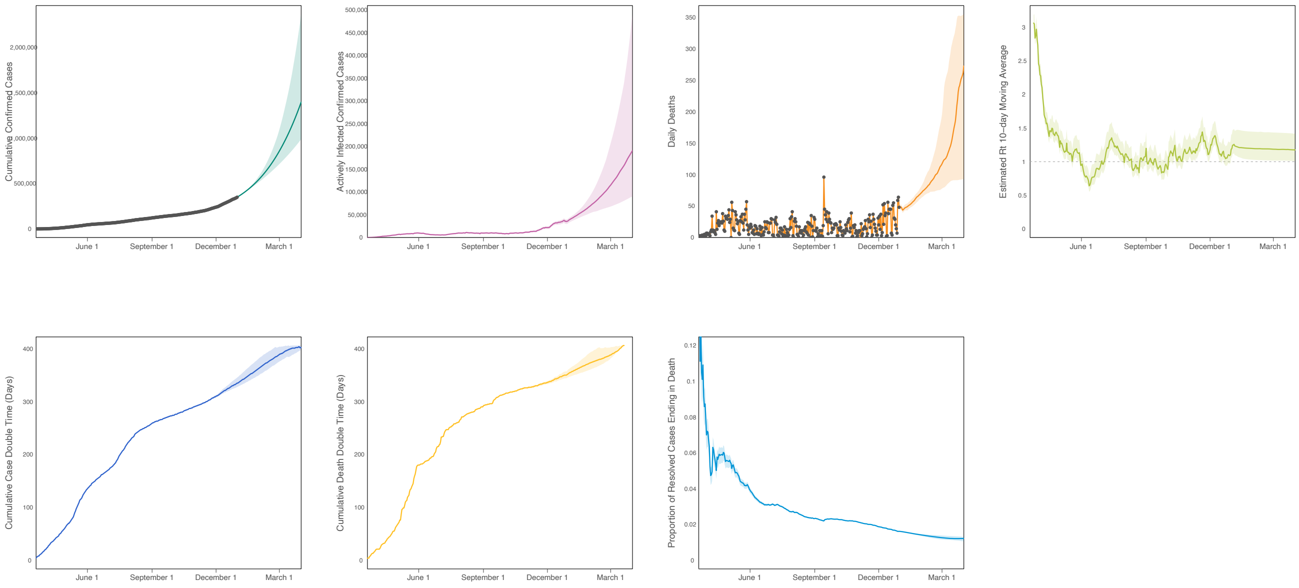
Cumulative Confirmed Cases
Actively Infected Confirmed Cases
Daily Deaths
Estimated Rt 10-day Moving Average
Cumulative Case Double Time (Days)
Cumulative Death Double Time (Days)
Proportion of Resolved Cases Ending in Death
June 1
September 1
December 1
March 1

WA

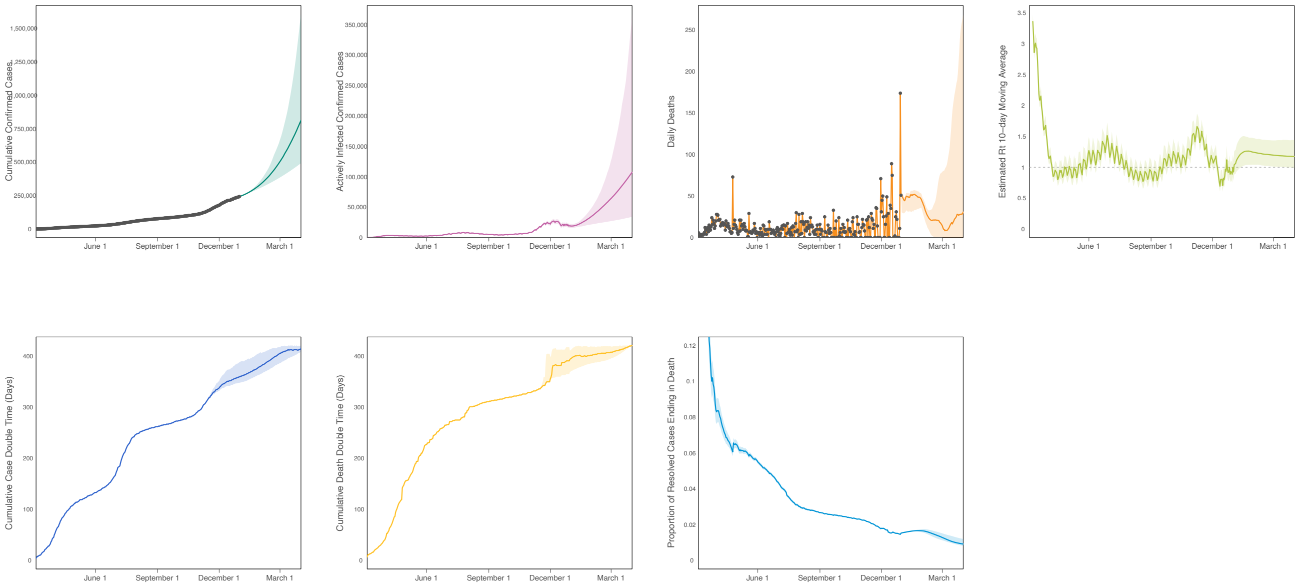
Cumulative Confirmed Cases
Actively Infected Confirmed Cases
Daily Deaths
Estimated Rt 10-day Moving Average
Cumulative Case Double Time (Days)
Cumulative Death Double Time (Days)
Proportion of Resolved Cases Ending in Death
June 1
September 1
December 1
March 1

WI

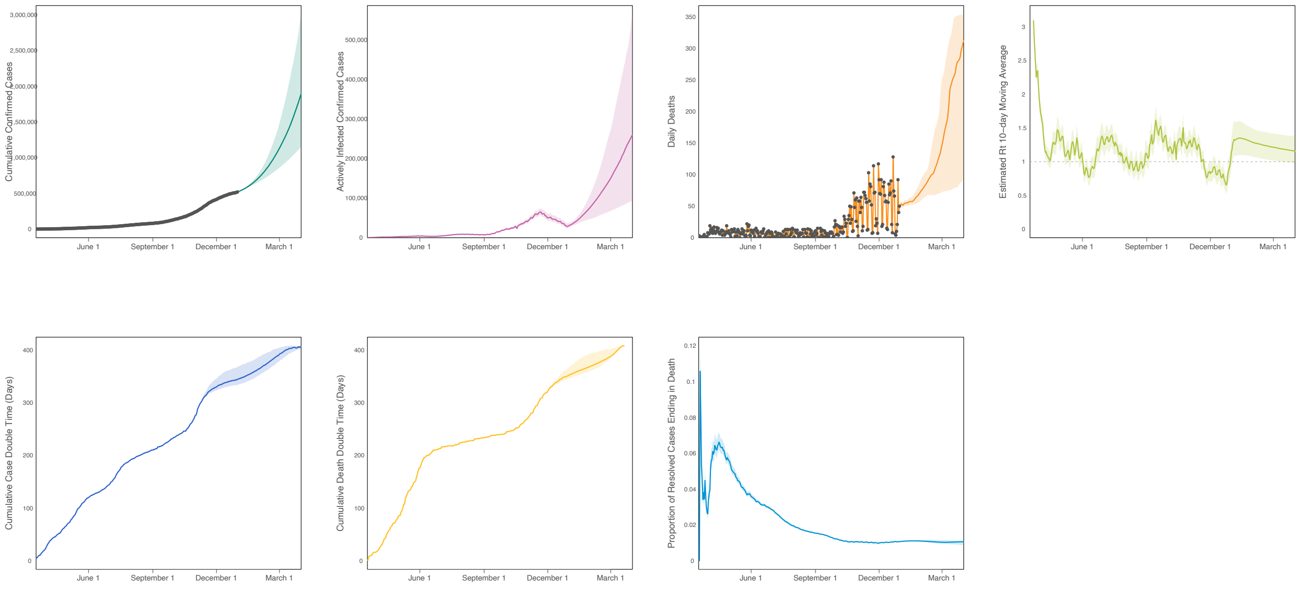
Cumulative Confirmed Cases
Actively Infected Confirmed Cases
Daily Deaths
Estimated Rt 10-day Moving Average
Cumulative Case Double Time (Days)
Cumulative Death Double Time (Days)
Proportion of Resolved Cases Ending in Death
June 1
September 1
December 1
March 1

WV

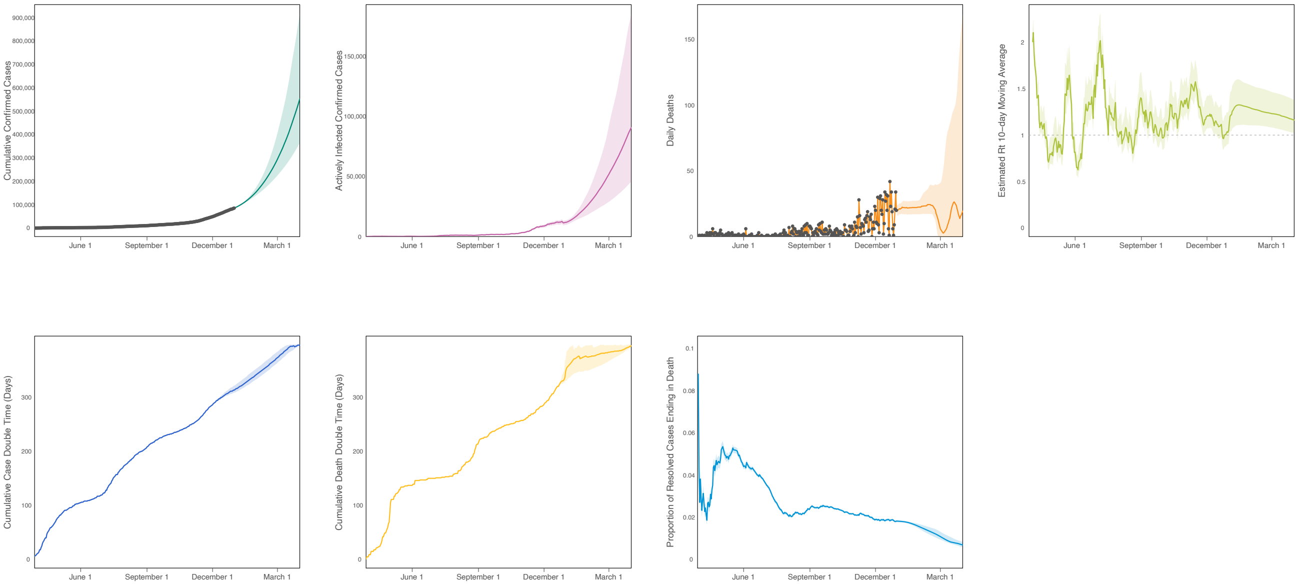
Cumulative Confirmed Cases
Actively Infected Confirmed Cases
Daily Deaths
Estimated Rt 10–day Moving Average
Cumulative Case Double Time (Days)
Cumulative Death Double Time (Days)
Proportion of Resolved Cases Ending in Death
June 1
September 1
December 1
March 1

WY

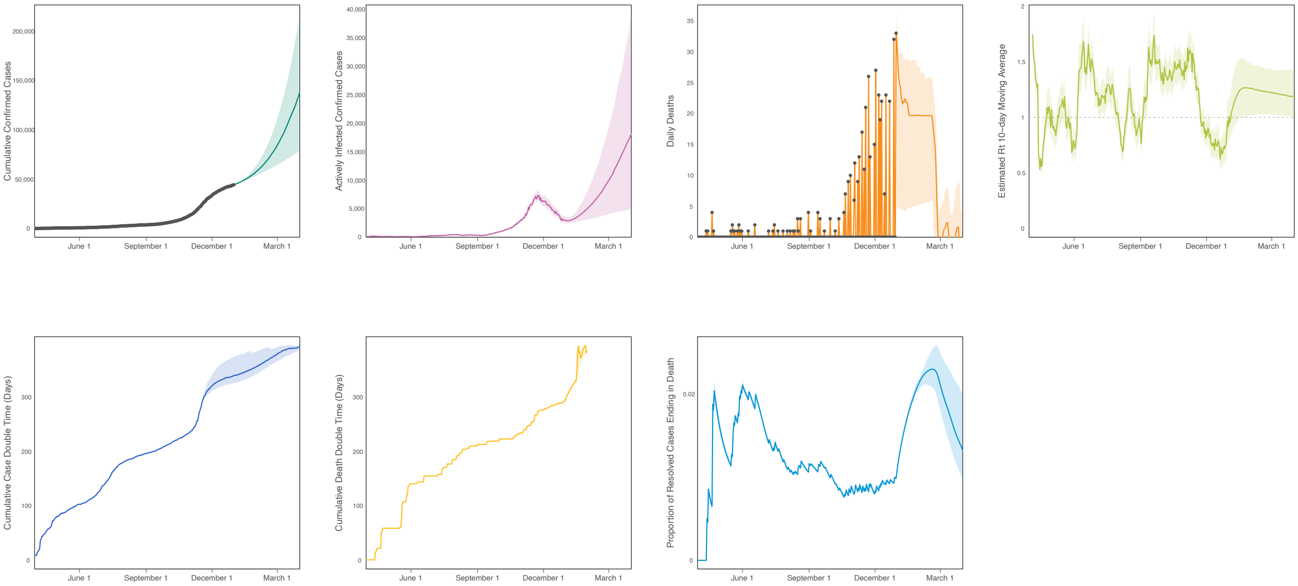
Cumulative Confirmed Cases
Actively Infected Confirmed Cases
Daily Deaths
Estimated Rt 10–day Moving Average
Cumulative Case Double Time (Days)
Cumulative Death Double Time (Days)
Proportion of Resolved Cases Ending in Death
June 1
September 1
December 1
March 1
