# Supplementary material for: Pandemic velocity: Forecasting COVID-19 in the US with a machine learning & Bayesian time series compartmental model
Source: PLoS Comput Biol. 2021 Mar 29;17(3):e1008837. doi: 10.1371/journal.pcbi.1008837 (PMC8031749; doi:10.1371/journal.pcbi.1008837)
Supplement: S1 Table — The parameter values and prior distributions for the parameters of each component of the model. (PDF) [file pcbi.1008837.s003.pdf]

Pandemic velocity: forecasting COVID-19 in the U.S. with a machine learning & Bayesian time series compartmental model, supplemental material

Gregory L. Watson[1], Di Xiong[1], Lu Zhang[1], Joseph A. Zoller[1], John Shamshoian[1], Phillip Sundin[1], Teresa Bufford[1], Anne W. Rimoin[2], Marc A. Suchard[1,3], Christina M. Ramirez[1]

1 Department of Biostatistics, Fielding School of Public Health, University of California, Los Angeles, California, United States of America
2 Department of Epidemiology, Fielding School of Public Health, University of California, Los Angeles, California, United States of America
3 Departments of Computational Medicine and Human Genetics, David Geffen School of Medicine, University of California, Los Angeles, California, United States of America

Table S1. Parameter Values & Distributions

Model	Parameter	Value/Distribution
SIRD	ρ^{-1}	$N(10, 1)$
Velocity Model	μ_μ	$N(0, 0.1)$
Velocity Model	μ_ϕ	$U(0, 1)$
Velocity Model	μ_τ	$Gamma(0.001, 0.001)$
Velocity Model	σ_μ^2	0.01
Velocity Model	σ_ϕ^2	0.05
Velocity Model	σ_τ^2	1
Velocity Model	`n.iter`	500,000
Velocity Model	`n.burnin`	10,000
Velocity Model	`n.thin`	1500
Random Forest	`ntree`	500
Random Forest	`mtry`	9
Random Forest	`nodesize`	5

Values and prior distributions for the parameters of each component of the model. The ρ parameter governs flow out of the I compartment of the SIRD model. Its inverse, ρ^{-1}, is interpreted as the expected amount of time until an infected individual recovers or dies.
