# Supplementary material for: Pandemic velocity: Forecasting COVID-19 in the US with a machine learning & Bayesian time series compartmental model
Source: PLoS Comput Biol. 2021 Mar 29;17(3):e1008837. doi: 10.1371/journal.pcbi.1008837 (PMC8031749; doi:10.1371/journal.pcbi.1008837)
Supplement: S2 Table — A comparison of the random forest death model to a state-specific autoregressive model over 4 different training and evaluation sets. (PDF) [file pcbi.1008837.s004.pdf]

Pandemic velocity: forecasting COVID-19 in the U.S. with a machine learning & Bayesian time series compartmental model, supplemental material

Gregory L. Watson[1], Di Xiong[1], Lu Zhang[1], Joseph A. Zoller[1], John Shamshoian[1], Phillip Sundin[1], Teresa Bufford[1], Anne W. Rimoin[2], Marc A. Suchard[1,3], Christina M. Ramirez[1]

1 Department of Biostatistics, Fielding School of Public Health, University of California, Los Angeles, California, United States of America
2 Department of Epidemiology, Fielding School of Public Health, University of California, Los Angeles, California, United States of America
3 Departments of Computational Medicine and Human Genetics, David Geffen School of Medicine, University of California, Los Angeles, California, United States of America

Table S2. Death Model Comparison

Final Day of Training Data	Random Forest	AR-1
August 31, 2020	7.27	7.58
September 30, 2020	8.31	7.22
October 31, 2020	11.4	12.35
November 30, 2020	18.19	21.06

The median state mean absolute error (MAE) for the random forest death model and a state-specific AR-1 model over the 21 days subsequent to the last day of the training data. The random forest death model performed better on 3 of the 4 evaluation periods. The greater MAE for later months is likely a result of the number of deaths increasing substantially over the course of the pandemic.
