# Supplementary material for: Pandemic velocity: Forecasting COVID-19 in the US with a machine learning & Bayesian time series compartmental model
Source: PLoS Comput Biol. 2021 Mar 29;17(3):e1008837. doi: 10.1371/journal.pcbi.1008837 (PMC8031749; doi:10.1371/journal.pcbi.1008837)
Supplement: Attachment — Submitted filename: Response to Reviewers.pdf [file pcbi.1008837.s005.pdf]

Seth Flaxman, Ph.D.
Faculty of Natural Sciences, Department of Mathematics
522 Huxley Building
Imperial College London
South Kensington Campus
London SW7 2AZ

August 31, 2020

Dear Dr. Flaxman and anonymous reviewer:

Thank you for taking the time to consider our manuscript and offer insightful feedback and for the opportunity to resubmit a revised manuscript. Your suggestions have been helpful and strengthened the paper. The most significant changes to the manuscript are:

1. Updated forecasts using training data through August 30;
2. Expanded and updated analysis of predictive accuracy;
3. Reorganized presentation of the model and its components to better motivate the individual components and clarify their integration.

A variety of additional changes and expanded discussions were incorporated to address the concerns and suggestions offered in your comments. Please find a detailed response to each comment below.

Sincerely,

Gregory L. Watson
Corresponding Author
Department of Biostatistics
UCLA Fielding School of Public Health
650 Charles E. Young Dr. South
Los Angeles, CA 90095-1772
gwatson@ucla.edu

Reviewer 1

Comment: *The method proposed by Watson and colleagues has various compelling aspects–it combines a Bayesian hierarchical model, compartmental SIRD model and a machine learning methods for forecasting into the future–with the goal of accurately forecasting COVID-19-confirmed cases and deaths. The model is meant to predict so-called "case velocity" and yields forecasting intervals, which is very important, and I am not concerned by the fact that they cannot be formally interpreted as posterior uncertainty intervals. Overall, while I think that the approach taken is a sensible one, there are a number of concerns and suggestions that I would make to the authors towards a much stronger revised version of the paper.*

1. Each of the modeling components of your method has its strengths and weaknesses. The strengths of the methods are not explained or utilized to the fullest degree possible; the weaknesses should be more fully explored through sensitivity analyses. I will taken each component in turn.

a. The strengths of the SIRD model are that it is mechanistic, however this is not exploited to, e.g. simulate various scenarios for opening up, etc. Estimates of the rate of transmission by state are also of particular importance—and can be compared to the estimates derived by other methods—but it does not seem that these are explicited reported anywhere unless I have missed them.

Reply: One of the benefits of our SIRD model is that it can be used to simulate alternative scenarios to provide insight to decision makers. However, detailing the additional data and assumptions required to do so would substantially expand the scope of this manuscript. Unlike more simplistic compartmental models in which alternative scenarios are often modeled simply by inflating or deflating a rate parameter, our model allows for time-varying adjustments. We think this complexity allows for more realistic scenarios, but there is a corresponding increase in the amount of discussion and presentation required to justify such an analysis. Because our modeling approach is non-traditional—we know of no other compartmental models that employ a velocity model—we focus here on justifying the methodological underpinnings of our modeling framework, and leave the presentation of alternate scenarios to future companion pieces. We have suggested this as an avenue for future work in the discussion, "This modeling framework suggests a number of avenues for future work. The most salient of these is the simulation of various scenarios that model policy or public health responses to the pandemic. Forecasting COVID-19 cases and deaths under alternate scenarios may provide useful information for decision makers."

Along with forecasts of cases and deaths, we do provide estimates of the transmission rate in the form of the effective reproductive number, R_t. While estimates of the reproductive number are hampered by the well-known challenges surrounding estimating the true number of COVID-19 infections in the US, they are still useful for gauging the rate of disease spread.

Comment: *A limitation of the SIRD model is that it requires setting initial conditions—your discussion on lines 120-123 should be expanded to understand the sensitivity our your approach to these choices. I am not an expert on SIRD, but I imagine as with any mechanistic model there are potentially many sensitivities to somewhat arbitrary choices. These should be explored.*

Reply: The initial conditions of a compartmental model strongly influence its forecasts, and our SIRD model is no exception. Our approach combines the available case data with the death and recovery portions of the SIRD model to construct a unique initial

condition for each SIRD model run. (There is one run for each posterior sample of the case velocity model.) Our motivation was to use the case data for all observed time points while capturing the uncertainty associated with recovery and death. We do not consider measurement error in the case data, which is a substantial limitation for COVID-19 data in the US and relates directly to our discussion of comment 4. To motivate this approach we have expanded this section to read as follows:

> A unique initial condition was constructed for each run of the compartmental model by stepping the model through each day of the observed data and fixing the number of cases to the observed value while using the recovery and death transition functions to distribute cases into compartments I, R and D. This combines the observed case data while attempting to account for the uncertainty in the number of individuals in I, R, and D using the randomness in the recovery and death functions. Using the observed case data and incorporating uncertainty reduces the sensitivity of the model to the choice of initial conditions. This approach ignores any measurement error in the case data, which is a substantial limitation considering the status of COVID-19 data in the US, as discussed above.

Comment: *b. The strengths of the hierarchical model are that it gives interpretable state-level estimates for parameters that policymakers care about. I saw little discussion of the posteriors over these parameters, how they compare to previous studies, etc. There are of course limitations here, which could be discussed as well, but I don't expect this to drastically change your overall results in any way.*

Reply: The hierarchical model provides state-specific forecasts for the velocity, which can be converted into forecasts of case growth. The parameters of the velocity model are not directly comparable to the transmission rate at the heart of more traditional compartmental models. Rather, they provide a mechanism for generating forecasts, which we use to estimate the effective reproductive number, $R(t)$. This and the predicted numbers of cases and deaths are natural points of comparison to other forecasts.

Comment: *c. The strengths of the random forest model are that it is an effective black box machine learning method that can use whatever covariates you throw at it. The limitations are that it is only partially interpretable—variable importance is useful, but does not give effect sizes. It would be very useful to know if a simpler, interpretable method were able to give reasonable results. Two ideas that you may have considered and would be worth a quantitative comparison: time series methods (e.g. autoregressive or exponential smoothing); carrying forward the SIRD model*

Reply: We agree with this characterization of the strengths and limitations of random forest. In our case, we are concerned only with predicting 1 day into the future and are not concerned with interpretability. We display random forest variable importance not for inference, but (1) to graphically depict the covariates to highlight them to the reader, and (2) to show the greater importance of lagged case counts, which conforms to our expectations. We have added a paragraph to the death model section detailing the reasons we selected random forest:

> We selected random forest for the death model over alternatives such as time series models, for 4 reasons: (1) in this context, we care only about predicting deaths given recent cases and other covariates rendering the interpretive and inferential advantages of time series models moot; (2) the

flexible mean structure of random forest accommodates nonlinear effects, interactions and provides implicit variable selection, all of which are much more challenging in a time series context; (3) each death model prediction is only one day into the future, not an entire time series; and (4) the relationship between cases and deaths appears to have shifted in the U.S. throughout the course of the pandemic so far (for reasons that are not entirely clear—increased testing, better treatment protocols, a younger infected population, and viral attenuation may be contributing factors), suggesting that a nonstationary time series model would be needed, making the process of fitting such a model even more challenging.

It may be possible to construct a time series model that rivals the predictive performance of random forest for our purposes, but we are doubtful, and devising such a model would be a challenging project in its own right and beyond the scope of the current work. To improve our random forest death model, we have added interval estimation using the procedure presented by Zhang et al. that produces prediction intervals from the out-of-bag errors (Zhang et al., 2019).

Comment: *2. Your SIRD formulation is non-standard, as far as I am aware. I would have expected theta(t) to appear in the equation for dI/dt. Is your Eq (2) somehow equivalent to the following, which is what I expected?*

dS/dt = -xi(t)
dI/dt = xi(t) - rho I (t) - theta(t)
dR/dt = rho I(t)
dD/dt = theta(t)

Reply: We have parameterized these equations slightly differently, because we have data on deaths but not in general on recoveries. (Some US states have reported recoveries, but in most instances reported recoveries appear to be limited to hospitalized patients who have recovered.) Consequently, we have focused on modeling deaths using the random forest death function ($\theta(t)$) and use ρ as an admittedly somewhat crude model for the rate at which individuals either die or recover. Thus $\rho I(t)$ becomes the expression for the total number of individuals moving out of I, of whom $\theta(t)$ die, i.e., $dD/dt = \theta(t)$, with the remainder of the $\rho I(t)$ recovering, i.e., $dR/dt = \rho I(t) - \theta(t)$. In essence we have split the R compartment of a traditional SIR model into R and D, with $\theta(t)$ defining the partition. To clarify this point, we have revised the discussion of these equations so that it now reads,

> Traditional SIR compartmental models use a rate parameter, which we call ρ, that is the inverse of the time an individual is expected to be infected to model the movement of individuals out of the infectious compartment. We follow this approach, but split the R compartment into R and D, because we have reliable data on COVID-19 deaths, but not on recoveries. (Some states have reported recoveries, but in most instances this is limited to hospitalized patients who have recovered.) Like a traditional SIR model, we let $\rho I(t)$ denote individuals exiting the infectious compartment, which corresponds to the $-\rho I(t)$ term in $dI(t)/dt$. Using CDC guidelines on home isolation as an estimate of the average time from testing positive to recovery or death, we sample ρ^{-1} for each run of the compartment model from a Gaussian distribution with mean 10 and standard deviation 1 [108]. The death model, $\theta(t)$, indicates how many of these die, i.e., $dD(t)/dt = \theta(t)$, with the remainder of the $\rho I(t)$ recovering, i.e., $dR(t)/dt = \rho I(t) - \theta(t)$.

Comment: *3. I am not totally convinced by how the three parts of your model fit together.a) It would be nice if there was more integration between the pieces—there are methods for Bayesian inference of SIR models using modern probabilistic programming languages like Stan. Could you have combined the hierarchical model with the SIRD model? I will not claim this is easy as I have not tried, but it would be good to discuss this.*

Reply: It is possible, in theory, to integrate the velocity model with the SIRD model, but this is not trivial. We are in the process of a collaborative effort to build such a model, taking an approach along the lines of Abdalla et al., 2019. It is less clear that the random forest death model can be incorporated, but using BART or a similar Bayesian approach, as another reviewer suggested, may be a path forward here. We have added a sentence to the discussion section suggesting this as an avenue of future work.

Abdalla, N., Banerjee, S., Ramachandran, G. and Arnold, S., 2020. Bayesian State Space Modeling of Physical Processes in Industrial Hygiene. *Technometrics*, 62(2), pp.147–160.

Comment: *b) the discussion surrounding Eqs 8-9 says that c_i is not identifiable and you find it by minimizing MSE, but I am not sure what c_i is and would like to know how well this minimization works and what effect it has. I'm also not convinced that this yields a posterior distribution for c_i, but given that ultimately you are not doing Bayesian inference it probably doesn't matter.*

Reply: The velocity regression model provides estimates for the velocity (first derivative with respect to time) of log cumulative cases at future time points, i.e., $d \log u(t)/dt$. In the compartmental model, we need an expression for $dS(t)/dt$, which equals $-du(t)/dt$. Solving for cumulative cases requires integrating the derivative above, which results in a constant, which we call c_i (i indexes location). The assumption made by the velocity model that $E \log(d \log S(t)/dt) = a_i t + b_i$ gives an expression for $dS(t)/dt$ that depends upon this unknown constant.

The velocity model on its own does not tell us a value for this constant. (Intuitively this is because knowing the derivative of a function alone does not tell you the value of the function, e.g., knowing $df(0)/dt = 3$ does not give the value of $f(0)$.) In an attempt to remain empirically grounded in choosing a value for c_i, for each posterior sample of the velocity model we pick the value for c_i that minimizes the MSE of the resulting $\hat{S}(t)$ compared against the observed data. It is admittedly unusual to think of the values for c_i as posterior samples, and since it indeed does not matter for our purposes, we have removed this claim. We have updated our discussion to more clearly explain this procedure. It now reads as follows:

> The lognormal model assumes the expectation of $d \log u_i(t)/dt$ is $\exp(a_i + b_i t)$. The compartmental model requires an expression for $du_i(t)/dt$, which can be obtained by integrating, solving for $u_i(t)$, and taking its derivative. The integration step introduces an additional parameter c_i (the integration constant), and
>
> $$u_i(t) = \exp\left(\frac{1}{b}_i \exp(a_i + b_i t) + c_i\right). \quad (1)$$
>
> While the MCMC procedure described above provided samples from the posterior distributions of a_i and b_i, it does not uniquely identify c_i. This is simply a result of integrating: the value of a function cannot be deduced from its derivative alone, e.g., knowing $df(x)/dx = 0$ for some function f

> does not determine $f(x)$. To remain empirically grounded while obtaining an expression for $u_i(t)$, we use the observed case counts to empirically estimate $c_i^{(m)}$ by minimizing a squared loss function defined over the observed cumulative count at location i for each posterior sample...

Comment: *c) I am trying to make sense of sampling rho ∼ Normal(14,1). Given your formulation of SIRD (which I questioned earlier), rho is the probability of transitioning from Infection to either Recovered or Death. You should provide citations to justify a mean of 14. I am familiar with estimates of the infection-to-onset distribution and the onset-to-death distribution, but this is neither of those.*

Reply: As mentioned above in response to comment 2, we use ρ in the manner of traditional S(E)IR models to model transition out of the I compartment. In this context it is the interval of time from infection (testing positive in our case) to either recovery or death, which are indistinguishable in a traditional S(E)IR model. This traditional approach is simplistic, but without reliable data on recoveries we thought it a reasonable strategy, and in line with many compartmental models of COVID-19. Since we do have data on deaths, however, we use a death model to predict how many of these individuals die. We have changed the mean of this distribution to be 10, based on updated information and CDC quarantine guidelines, which we have cited:

> Like a traditional SIR model, we let $\rho I(t)$ denote individuals exiting the infectious compartment, which corresponds to the $-\rho I(t)$ term in $dI(t)/dt$. Using CDC guidelines on home isolation as an estimate of the average time from testing positive to recovery or death, we sample ρ^{-1} for each run of the compartment model from a Gaussian distribution with mean 10 and standard deviation 1 [108].

Comment: *d) The random forest component of your approach is meant to give an estimate of the transition from infection to death, but I am not convinced that this is what it is truly doing due to my concerns about the reliability of case reports (see below). To understand and diagnose this concern, it would be helpful to compare your random forest model's predictions of the number of people transitioning from I to D to published estimates of the infection fatality rate. Admittedly, I'm a little unclear on whether/how these can be directly compared, so if this is impossible it would be useful to know why as I see this as a limitation of your model, despite it having an underlying epidemiological component. Returning to the infection-to-death distribution, estimates put this at around 2 to 3 weeks, so it seems like your random forest model should include data that is more than 14 days in the past. (In fact, I don't see what is the relevance of data t-1 or t-2!) The data limitations you mention can now be alleviated as I imagine you will want to refit your model on the most recently available data.*

Reply: We have extended the lagged case data covariates to 21 days. There was no appreciable improvement in out-of-bag R^2 beyond this. We have retained cases at $t-1$ and $t-2$ as covariates, even though it would be unusual for individuals to die 1 or 2 days after testing positive, because our interest is in prediction not inference. It may also be possible that some individuals are not tested for COVID-19 until they are severely ill, particularly if they are uninsured and had not sought treatment previously.

Comment: *4. The reliance on cases concerns me with respect to accurately modeling COVID-19 due to the widespread, undetected community transmission in the early period of the USA epidemic, the changing testing strategies and availability during the course of the USA epidemic, and the large fraction of asymptomatic cases throughout.*

Attempts have been made to adjust for some of this (e.g. Hsiang et al, Nature 2020) but I am not convinced that this is truly possible, which is why my group has focused on modeling deaths instead of cases (Flaxman et al, Nature 2020). This all leads to another important concern, which is that your overall goal is predict case velocity. I can see this as potentially a useful goal, especially in the post-stay-at-home phase of the epidemic and the possible second wave phase of the epidemic, but would like to see more justification for this goal. I am still not convinced that case reporting is good enough that we should rely on it—if it is not good enough, then why is it your goal to predict it? Said differently, perhaps you wish to predict the true rate of cases; but then what, exactly is a case? The true thing that you could try to predict is infections. I am certainly not saying you need to take the approach of our group, but for work in this vein see Flaxman et al Nature 2020 and our US report: https://www.imperial.ac.uk/media/imperial-college/medicine/mrc-gida/2020-05-28-COVID19-Report-23-version2.pdf

Reply: The substantial fraction of COVID-19 infections that are asymptomatic combined with the limited availability and use of testing in the US, especially early in the pandemic, certainly makes modeling case data challenging at the very least. Modeling deaths is an appealing alternative and worthwhile direction. The primary advantage to modeling cases is they provide up-to-date information on the spread of infections, whereas deaths lag infections. We have added a "Data" subsection to the "Materials and methods" section to thoroughly articulate our motivation in using the case data, despite the challenges.

The velocity model provides an empirical approach to forecasting case growth. We forecast new COVID-19 cases by modeling the velocity of the log cumulative cases. Forecasting COVID-19 cases in this velocity domain is appealing, because it reveals seemingly subtle shifts in case trajectory that are not obvious when considering raw case counts. We have elaborated upon the virtues of this approach in the velocity model subsection (now entitled, "Bayesian Velocity Model for Forecasting Cases"):

> Let $u_i(t)$ denote the cumulative case count for location i at time t. The velocity (the first derivative with respect to time) of the log transformed cumulative cases is the instantaneous rate of new cases to cumulative cases at a given time,
>
> $$\frac{d}{dt}\log u_i(t) = \frac{du_i(t)}{dt} \cdot \frac{1}{u_i(t)},$$
>
> which is related to the reproductive number, but is readily estimated from the data.
>
> Calculating the reproductive number at a particular time, on the other hand, requires knowing the number of active infections. There is currently no reliable data on this, as most infections resolve on their own outside of a clinical or otherwise supervised setting in which their transition from active case to recovered might be recorded.

Comment: *5. If the overall goal is accurate forecasting of case velocity, then I think there are two pieces missing: a. A more indepth and exhaustive approach to evaluating your forecasting method—this could, e.g. include 1, 3, 7, and 21 day ahead forecasting, starting from an early period in the epidemic and carried forwards with subsequently more days of data included. Various metrics could be included, including MAE/coverage/CRPS.*

Reply: We have expanded our evaluation of predictive accuracy, by training the model through the 30th of each month from April through July and projecting forward for 21

days. Figure 4 in the manuscript now depicts the median and interquartile range of the prediction error (MASE) for cases and deaths at each day in the 3-week forecast evaluation period. For both cases and deaths, the median MASE was well below 1 for 21 days past the end of the training data in all 4 instances, an encouraging sign for the reliability of our predictions.

Comment: *b. A justification for including the SIRD and hierarchical modelling framework; would a black box machine learning method on its own give at least as good performance as your method? If so, what is it that your model reveals about the spread of the disease that a black box model on its own would not?*

Reply: There are several advantages of our approach over a black box machine learning method. First, the SIRD model provides a joint model for cases, deaths and recoveries, which most black box prediction tools would not provide. Second, the hierarchical model provides full uncertainty quantification for the velocity model, which is not straightforward for many black box prediction tools. Typically cross-validation (CV) or bootstrap would be used for this, but the time-dependent structure of the data combined with the need for state-specific predictive trajectories complicates resampling strategies. We propagate uncertainty through the compartmental model by running it for each posterior sample of the hierarchical model. Third, the velocity model enables case forecasting in a way that would be difficult with many black box tools, since this prediction is out-of-domain. We have added mention of these advantages when introducing the SIRD model:

> The compartmental model allows for the joint forecasting of these quantities, a distinct advantage over many approaches including so-called black box prediction tools that generally only model a single outcome. The posterior samples from the velocity model provide a mechanism for uncertainty quantification that can be propagated through the compartmental model. The compartmental model also allows the case forecast to be used as covariates in the death model, which otherwise would not provide predictions beyond one day past the observed data.

Comment: *6. Source code to replicate every part of your analysis should already be made available. In this world of falling public trust in scientists and policymakers, it is critically important to strive for replicable and reproducible analyses. The effort spent is entirely worth it due to the benefit that comes from having more pairs of eyes inspecting your assumptions, your model, your data pipeline, and so on.*

Reply: We have made R code available at https://github.com/gregorywatson/covidStateSird.

Comment: *7. Speaking of data, I may have missed it but do you use JHU or NYT data? We found that JHU data was on the whole reliable, though it suffers from various inconsistencies, and it is important to point out that this data is usually date of report for deaths, rather than date of death. (The JHU data was not reliable for New York State, and for this we used NYT.)*

Reply: We use data made available by The COVID Tracking Project at https://github.com/COVID19Tracking/covid-public-api. We have added a “Data” subsection to the “Materials and methods” section to more thoroughly articulate our motivation in using case data, despite its challenges. This subsection includes the following sentence that identifies the source data we use, “Daily COVID-19 confirmed

cases and deaths for each state were obtained from the COVID Tracking Project, which combines information from state health departments and other sources."

Comment: *8. A minor issue, and I am sorry to quibble about this but it is of course the part I know best: I do not agree with your characterisation of the Flaxman et al model as a serial growth model (honestly I'm not sure what this means so maybe I'm wrong!), nor is the description of "weights being sampled from a probability distribution" an accurate description of our Bayesian inference method (perhaps it is an accurate description of the other methods). Compare, for example, our fully Bayesian MCMC scheme to the number of infections on a given day (which derives from a discrete time convolution using the generation distribution multiplied by the time-varying reproduction number R) to your scheme for introducing stochasticity into your SIRD model by sampling rho which could accurately be described as weights being sampled from a probability distribution.*

Reply: Thank you, we agree and have corrected this in the introduction.

Reviewer 2

Comment: *In this paper, "Fusing a Bayesian case velocity model with random forest for predicting COVID-19 in the U.S." by Watson et al. (2020), the authors propose an approach to forecasting mortality forecasts for the ongoing COVID-19 pandemic in the United States by fusing regression models with compartment models. This method is validated using a holdout sample of cases and deaths data, and used to make forecasts for future unobserved cases and deaths.*

1. Overall, I think this paper provides some innovative methodology which potentially constitutes a meaningful contribution to statistical methodology and epidemiological forecasting. However, I have some considerable reservations regarding particular modeling choices, the soundness of using case count data in forecasting, and the presentation. I outline these concerns below.

Major comments

Comment: *2. I frankly get lost in the presentation of the paper. It starts out presenting the compartment model, and quickly dives into the regression models. This all feels unmoored. The paper would be well served by better internal signposts guiding the reader to the overall modeling approach of the paper. How exactly do all the pieces fit together?*

Reply: We have restructured the "Materials and Methods" section to better organize our presentation of the components of the model and how they are integrated. There are now six subsections:

1. Data
2. Model Overview
3. Velocity Model
4. Death Model
5. SIRD Model
6. Predictive Accuracy

The Model Overview subsection lays out and motivates the overall approach, before the subsequent sections on the individual components articulate the details of each. In brief,

the velocity model and the death model each become transition functions within the SIRD compartmental model:

> There are three primary components to our model: (1) the case model for predicting new confirmed cases, (2) the death model for predicting how many cases end in death, and (3) a four compartment epidemiological model that fuses these together to provide joint predictions of cases, deaths and recoveries. The case model and the death model become transition functions within the compartmental model. There are several advantages to this combined approach. First, the SIRD model provides a joint model for cases, deaths and recoveries, allowing simultaneous forecasting of these. This is an advantage over univariate models, including statistical regression models and machine learning prediction tools, which can only forecast one outcome. Second, the combined approach incorporates information on projected case growth into death predictions in a very flexible manner, which would not be available if the models were separate or if a less flexible death model were used. Third, the velocity model for projecting case growth both fits the observed data and extrapolates well, which is a challenge for curve-fitting approaches. Fourth, we incorporate uncertainty of model fit into the compartmental forecasts, by running it many times–once for each posterior sample from the Bayesian case model.

We have also elaborated upon the motivation for each component and its place within the overall framework at the beginning of the velocity, death and SIRD model subsections to present them within the context of the overall approach and to make a more convincing case for our modeling choices.

Comment: *3. The main compartment model used for the paper is given in by the set of differential equations in Esq. (2). Because the final prediction are so heavily informed by this compartment model, its structure should be explained and its use justified. For instance, I have seen the use of many such models where the equation* $dI(t)/dt$ *includes a term involving* $\beta S(t)I(t)/N$ *for population* N *and some* $\beta > 0$*, because the number of people becoming newly infected depends on people currently infected infecting those who are susceptible. See Diekmann et al. (2010) and the references therein for some examples.*

Reply: Compartmental models traditionally include a term involving $\beta S(t)I(t)/N$ or just $\beta S(t)I(t)$ for $dI(t)/dt$. We found this functional form for $dI(t)/dt$ fit the observed data very poorly, and consequently we have replaced it with a more complicated function, which we call $\xi(t)$, derived from our case velocity model. Importantly, $\xi(t)$ does not depend on $I(t)$, which is a departure from traditional compartmental models and is similar to the approach of so-called curve-fitting models. This hybrid approach was motivated by a desire to retain the benefits of compartmental models while exploiting the substantially better empirical accuracy of curve-fitting models for the changing number of cases.

We have elaborated upon this point in the manuscript to make clear how and why our approach differs from traditional compartmental models:

> The transition between S and I is determined by $\xi(t)$, which describes the number of individuals becoming confirmed COVID-19 cases. This differs from traditional compartmental models. The standard expression for $dI(t)/dt$ is $\beta S(t)I(t)$ (sometimes divided by the population size N) as described in the introduction. We found the traditional functional form for dI(t)/dt fit the observed data very poorly, which motivated its

> replacement by $\xi(t)$, a time-varying function derived from our case velocity model. Importantly, $\xi(t)$ does not depend on $I(t)$, which is a departure from traditional compartmental models and is similar to the approach of so-called curve-fitting models. This hybrid approach was motivated by a desire to retain the benefits of compartmental models while exploiting the substantially better empirical accuracy of curve-fitting models for the changing number of cases.

Comment: *4. The authors model the number of COVID-19 cases. However, COVID-19 testing was notoriously scant in the US in the early stages of contagion there. This may explain why the case velocity is constantly decreasing; presumably if testing were more widely available early on in the pandemic, the velocity would increase then decrease. Testing remains inconsistent across states in terms of its availability and distribution. Therefore, use of this data is questionable and should be justified.*

Reply: The availability of COVID-19 testing in the US, especially early in the pandemic, was certainly woefully inadequate. This along with the substantial fraction of infections that are asymptomatic makes modeling case data challenging at the very least. We have added a "Data" subsection to the "Materials and methods" section to thoroughly articulate our motivation in using case data, despite these challenges.

While testing definitely impacts case velocity, which we would certainly expect to increase early in a COVID-19 outbreak, we model the velocity of log cumulative cases. A constant log cumulative case velocity corresponds to exponential case growth, so we would not necessarily expect to see increasing velocity of log cumulative cases early in an outbreak (although it is possible). More importantly, however, the number of cases early in an outbreak are small, making estimates of the velocity (of both cases and log cases) highly uncertain. In any case, we do not need to accurately estimate velocity during this initial period as the pandemic has moved irreversibly well past it at least in every state of the US, the domain over which we are interested in forecasting.

We have also expanded our discussion of the velocity of log cases to clarify this point in the first paragraph of the velocity model section.

Comment: *5. The authors claim that models which use time-varying predictors such as social media use or cell phone location data depend on data which is unknown to the future and so must make assumptions regarding these data. This may be true, but this model makes plenty of assumptions of its own! Of course, assumptions needed for any model, and should be commensurate with the intended use of the model.*

Reply: Every model indeed relies upon assumptions. We do not intend to denigrate models with different assumptions, we simply offer a word of caution regarding covariates that may be very informative in fitting historical data whose future values are unknown. Forecasting is difficult, and forecasting the course of an unprecedented event like the COVID-19 pandemic is very difficult. Incorporating these types of covariates is certainly appealing and can be quite useful, especially when there is good reason to be confident in one's prior beliefs about their future values. They are not, however, a panacea, and we hope to clarify that in our survey of modeling approaches for less technically savvy readers who may question any modeling effort that excludes them on the grounds of their predictive power for historical data. To articulate this more clearly, we have expanded the discussion at this point to read as follows:

> Time-varying covariates like mobile phone tracking data [91], Google trends [89, 92], and social media [93] are easily incorporated into such a model and may be quite predictive of the observed data. These data are

not a panacea, however, as forecasting requires knowledge of their values at future times, which are as yet unobserved. The forecasting accuracy of a model incorporating these covariates can depend heavily upon the accuracy of the assumptions made regarding their future values.

Comment: *6. One of the biggest assumptions is that the case velocity is linear depending on time t, with some shift in the slope on t after an intervention. However, my intuitive understanding is that the velocity should increase first and then decrease (at least if testing was widely available). In this case, ideally a quadratic term for time should be used. In either case, the model basically assumes that the case velocity will always go to 0. But this is certainly not true. The velocity will increase again if people revert to pre-social distancing behavior (which we are seeing in states in the West and South of the US as I write this), or at least if and when a second wave starts.*

The implications of the assumption embedded in the regression structure in Eq. (3) should be discussed. It should be acknowledged that, at best, this model is useful for predicting only cases and deaths of a single wave, assuming that the impact of policy interventions are constant across time with respect to decreasing infections.

Reply: We use a log linear model for the velocity of log cumulative cases. Constant velocity corresponds to exponential case growth, so we do not necessarily expect the velocity to be increasing early in an outbreak. Over time, the velocity of log cumulative cases generally decreases with time, because it is equivalent to the instantaneous rate of new cases to cumulative cases at a given time (see our response to comment 12). As cumulative cases are monotonically increasing, we expect the velocity to tend to decrease over time. Indeed with a finite population, the velocity must always go to 0 sooner or later, one way or another.

Of course it is possible for the velocity to increase as we have seen in certain parts of the US. However, in all cases so far this increase is temporary, and the velocity comes back down to whatever level of case growth the population tolerates. Increasing velocity has not been sustained, because it corresponds to exponential growth at an increasing exponential rate. This very rapidly results in an explosion of cases that has always triggered at least some level of shift in behavior or policy response to reduce the rate of case growth. While our velocity model does not predict when future waves or reversion to pre-social distancing behavior may occur, it accommodates these events fairly well, because these events rapidly push case growth above tolerable levels, and there is always a subsequent return to whatever velocity corresponds to the population's particular tolerance.

This is an important point, and we have added the following paragraph to the discussion:

> The linear mean structure of our lognormal velocity model limits its flexibility, which means that without the addition of covariates it cannot predict the occurrence of future waves of cases that correspond to an increasing velocity that may result, for example, from a return to pre-social distancing behavior. Our model does, however, accommodate these types of events quite well. Increasing velocity corresponds to exponential growth at an increasing exponential rate. This rapidly causes an explosion of cases that pushes case growth beyond whatever level a particular population tolerates. In every case there has been a subsequent return to a velocity that corresponds to a tolerable level of case growth. By targeting this velocity, our model forecasts reasonable long-term case trajectories without needing to predict the occurrence of case spikes, which are very difficult to anticipate.

Comment: *7. The authors correctly point out the importance of providing uncertainty in their forecasts. This justifies the use of a Bayesian model for the cases trajectory, but then the authors use random forest for the deaths model. With the understanding that computation becomes more difficult, it is possible to use a Bayesian model for deaths? For instance, Bayesian additive regression trees (BART; Chipman et al., 2010). See Dorie et al. (2020) for an easy-to-use implementation of BART in R in the dbarts package. You also use the bartMachine package (Kapelner and Bleich, 2016) for an implementation which allows reporting variable importance.*

Reply: To quantify the uncertainty associated with the random forest predictions in our death, we have incorporated the procedure devised by Zhang et al. that produces prediction intervals from the out-of-bag errors. This provides a 95% prediction interval for each run of the compartmental model. We use the fifth quantile of the distribution of interval lower bounds and the 95th quantile of the upper bounds to produce an overall prediction interval. We have added a description of this process to the manuscript, which reads,

> To quantify the uncertainty associated with random forest predictions, we follow the procedure devised by Zhang et al. to produce 95% prediction intervals from the out-of-bag errors [103, 104]. This results in a prediction interval for each run of the compartmental model. We take the fifth quantile of the distribution of interval lower bounds and the 95th quantile of the upper bounds to produce an overall prediction interval.

BART is certainly a potential alternative to random forest, and has the advantage of built-in uncertainty quantification. However, because of the increase computational burden, we have opted to continue using random forest. One potential avenue for future work is to combine the velocity model, compartmental model and death model into a unified Bayesian model. BART could be very useful to such an effort.

Comment: *8. The predictive accuracy results shown starting around line 263 are, in my opinion, not especially informative without any comparison to some other method or natural benchmark.*

Reply: MASE (mean absolute scaled error) does provide a natural benchmark of sorts by scaling the forecast error by the mean absolute error of naive, random walk in-sample prediction. We have expanded our evaluation of predictive accuracy, by training the model through the 30th of each month from April through July and projecting forward for 21 days. Figure 4 in the manuscript now depicts the median and interquartile range of the prediction error for cases and deaths at each day in the 3-week forecast evaluation period.

Minor comments

Comment: *9. I have often seen the derivatives in compartment models written as $\dot{S}(t)$ (i.e., \dot in LATEX) instead of $dS(t)/dt$. This notation may be convenient to use in this paper as well.*

Reply: Dot notation for derivatives with respect to time is commonly used in some fields, e.g., physics, and is certainly a reasonable choice for compartment models. We prefer to use $dS(t)/dt$ in this paper, however, because the sweeping impact of COVID has prompted interest in epidemiological models from researchers across many fields, and d/dt notation is very widely understood.

Comment: *10. The text size in the figures should be larger.*

Reply: We have increased the text size in each of the figures for improved legibility.

Comment: *11. The SIRD model in Fig. 1 would be improved if there were parameters written above the arrows which determine the transition rates between the compartments as written in Eq. (2). For instance, the arrow going from S to I should have an written above it.*

Reply: We have revised Figure 1 to include the transition functions from Equation 2 as suggested.

Comment: *12. Why model the "case velocity"? Why not just model the daily number of cases, which is essentially identical to case velocity as it is defined in the paper? Then one could use a count model (Poisson or negative binomial regression) for the case counts data, and still preserve the log-linear structure already used. The rstanarm package (Goodrich et al., 2018) has easy to use implementations of Bayesian count models.*

Reply: The daily number of new cases is indeed related to case velocity. However, we model the velocity of log cumulative cases, i.e.,

$$\frac{d}{dt} \log u(t) = \frac{du(t)}{dt} \cdot \frac{1}{u(t)}.$$

So this velocity is the instantaneous rate of new cases to cumulative cases at a given time, which is related to the reproductive number, but can be estimated directly from the data. If we were modeling $du(t)/dt$ then it would be quite similar to the daily number of cases, and perhaps a count model would be preferable. That is a reasonable approach that has been employed by other modellers. One of the goals of our work is to introduce velocity of log cases as an interesting alternative for others to consider in ongoing COVID-19 modeling efforts. Forecasting COVID-19 cases in this velocity domain is appealing, because it reveals seemingly subtle shifts in case trajectory that are not obvious when considering raw case counts.

To clarify this point in the paper, we no longer refer to our case model as a "case velocity model", which suggests that it could be a model for $du(t)/dt$, and refer to it simply as a "velocity model" or explicitly mention that it is a model for the velocity of log cumulative cases. We have also added a paragraph to the Velocity Model section to motivate this approach:

> We forecast new COVID-19 cases by modeling the velocity of the log cumulative cases. Forecasting COVID-19 cases in this velocity domain is appealing, because it reveals seemingly subtle shifts in case trajectory that are not obvious when considering raw case counts. Let $u_i(t)$ denote the cumulative case count for location i at time t. The velocity (the first derivative with respect to time) of the log transformed cumulative cases is the instantaneous rate of new cases to cumulative cases at a given time,
>
> $$\frac{d}{dt} \log u_i(t) = \frac{du_i(t)}{dt} \cdot \frac{1}{u_i(t)},$$
>
> which is related to the reproductive number, but is readily estimated from the data.
