# Supplementary material for: Pandemic velocity: Forecasting COVID-19 in the US with a machine learning & Bayesian time series compartmental model
Source: PLoS Comput Biol. 2021 Mar 29;17(3):e1008837. doi: 10.1371/journal.pcbi.1008837 (PMC8031749; doi:10.1371/journal.pcbi.1008837)
Supplement: Attachment — Submitted filename: PCOMPBIOL-D-20-00822R1-review.pdf [file pcbi.1008837.s006.pdf]

Review: PCOMPBIOL-D-20-00822R1

Anonymous

October 22, 2020

In this paper, "Fusing a Bayesian case velocity model with random forest for predicting COVID-19 in the U.S." by Watson et al. (2020), the authors propose an approach to forecasting mortality forecasts for the ongoing COVID-19 pandemic in the United States by fusing regression models with compartment models. This method is validated using a holdout sample of cases and deaths data, and used to make forecasts for future unobserved cases and deaths.

I applaud the authors for their improvements and appreciate the detailed way they address the comments I made on the first draft. Overall I believe the paper has been significantly strengthened now lays out an appealing fusion of epidemiological dynamics and statistics / machine learning methodology. However, I still have a few reservations which I detail below.

Comments

- I am somewhat more appreciative of the merits of modeling the "velocity" as the authors define it. The main merit seems to be that, compared to modeling the daily case numbers, it can handle underreporting by rather looking at the proportional daily increase in cases. However, I am still not fully convinced that the velocity model is the best measure. The model assumes that the velocity has independent Gaussian errors with the variance decreasing linear in time, due to the velocity being inversely proportion to the current cumulative number of cases. I must say that this a bit clumsy to me. One idea I had was modeling the log-growth rate. Let $v(t)$ be the daily number of cases one day t. The log-growth rate is

  $$y'(t) = \log \frac{v(t)}{v(t-1)}.$$

  This looks similar to the derivative of the cumulative case number, and like the velocity measure it is more robust to underreporting. However, with the growth rate it might not be necessary to shoehorn in the assumption of linearly decreasing variance.

- I agree with reviewer 1's comment no. 2 regarding the expression of the SIRD model. As the authors currently have it, it looks like subjects go into the R compartment before going to the D compartment. This is incoherent. Rather, as reviewer 1 says, the I compartment should branch out into R and D. The fact that R is unobserved is irrelevant to this fact.

- The ρ parameter in your SIRD model should correspond to estimates of the time from infection to recovery or death rather than what is recommended by CDC; for instance, see Verity et al. (2020): `https://www.thelancet.com/journals/laninf/article/PIIS1473-3099(20)30243-7/fulltext`

- I believe there is an equal sign missing in line 224, which should read: $dS_i(t)/dt = -du_i(t)/dt = -\xi_i(t)$

- I don't expect the authors to do this, but I think one ultimate goal could be constructing a joint model combining all these components, i.e. the log-linear model for cases, the tree model for deaths, and the differential equation system. This model would be "fully Bayesian" if you swap in BART for random forests.
