# Supplementary material for: Pandemic velocity: Forecasting COVID-19 in the US with a machine learning & Bayesian time series compartmental model
Source: PLoS Comput Biol. 2021 Mar 29;17(3):e1008837. doi: 10.1371/journal.pcbi.1008837 (PMC8031749; doi:10.1371/journal.pcbi.1008837)
Supplement: Attachment — Submitted filename: Response to Reviewers 2nd Round.pdf [file pcbi.1008837.s007.pdf]

Feilim Mac Gabhann, PhD
Department of Biomedical Engineering
Johns Hopkins University
720 Rutland Avenue
Baltimore, Maryland 21205

Jason Papin, PhD
Department of Biomedical Engineering
University of Virginia
Charlottesville, Virginia 22908

January 5, 2020

Dear Dr. Gabhann and Dr. Papin:

Thank you for again supplying us with reviewer feedback and providing us with the opportunity to submit a revised manuscript. The rewievers' suggestions have further strengthened our modeling effort in general and this manuscript in specific. The most significant changes to the manuscript are:

1. Updated forecasts using training data through December 31, 2020;
2. State-specific, time-varying forecasts of R_t, case doubling time, death doubling time and the proportion of cases resolving in subject death are now default outputs of the model and included with the figures in the supplemental material;
3. A revised velocity model that replaces the linear mixed model with an autoregressive model that better fits the data, is conceptually cleaner and computationally advantageous;
4. An improved death prediction model that now incorporates lagged death counts and is evaluated against an autoregressive prediction model.

Additional changes were incorporated to address other reviewer suggestions. Please find a detailed response to each comment below.

Sincerely,

Gregory L. Watson
Corresponding Author
Department of Biostatistics
UCLA Fielding School of Public Health
650 Charles E. Young Dr. South
Los Angeles, CA 90095-1772
gwatson@ucla.edu

Reviewer 1

Comment: *Dear authors,*

Thank you for your comprehensive response and set of revisions. In particular, your clarification and focus on "case velocity" has definitely sharpened the paper and its goals.

Reply: Thank you for this comment.

Comment: *There are a few final issues that I think you should address to strengthen your paper and its support for the new method you have proposed.*

1) I still think it would be very worthwhile to address this point: "The random forest component of your approach is meant to give an estimate of the transition from infection to death, but I am not convinced that this is what it is truly doing due to my concerns about the reliability of case reports (see below). To understand and diagnose this concern, it would be helpful to compare your random forest model's predictions of the number of people transitioning from I to D to published estimates of the infection fatality rate." If helpful, a meta-analysis on IFR is now available https://www.imperial.ac.uk/mrc-global-infectious-disease-analysis/covid-19/report-34-IFR/

Reply: To facilitate this comparison we now include the proportion of resolved cases that end in death as an output of the model by default. This proportion changes over time in both the observed data and in the model output and is graphically depicted for each state among the figures in the state predictions supplement. This proportion estimates the case fatality rate, and thus is higher than the infection fatality rate estimates of Brazeau et al. Nevertheless the estimate provides a useful point of comparison with other modeling approaches and also a depiction of how this rate has evolved over the pandemic and what it may look like in the future.

Comment: *2) You write: "The parameters of the velocity model are not directly comparable to the transmission rate at the heart of more traditional compartmental models". This clarification makes sense to me, but I do still wonder if you could include some direct comparisons to help the reader get a handle on how velocity works, by comparing it to more widely used (and I would argue better understood) quantities such as: the reproduction number, growth rate, or doubling time. Perhaps, for example, you could forward simulate from your model and then empirically calculate the doubling time?*

Reply: We now output estimates of R_t as well as the case and death doubling times with each model run to allow for direct comparison with the output of other models. Plots of these quantities are included with the other output for each state in the supplemental material.

Comment: *3) Your points beginning "We agree with this characterization of the strengths and limitations of random forest." make sense to me; thank you for the clarification. I would quibble with this part, however, "It may be possible to construct a time series model that rivals the predictive performance of random forest for our purposes, but we are doubtful, and devising such a model would be a challenging project in its own right and beyond the scope of the current work." Since your focus is on predicting one day ahead, I really think it would be helpful for you to include a very simple baseline comparison, such as an autoregressive model, trained separately for each state, or even a no change / constant model. If your model is significantly better than this model, great; if it is competitive, that is fine as well, as you have persuasively*

argued that there is something to be gained by focusing on velocity rather than simply predicting case counts.

Reply: As suggested, we have added a comparison of the random forest death model to an autoregressive model trained separately for each state. We evaluated the mean absolute error (MAE) of both models for each state over 4 different training and evaluation sets. The random forest death model performed better for 3 of the 4 evaluation periods. The details and results of this evaluation are included in section S4 of the supplemental material, and are discussed in the final paragraph of the "Predictive Accuracy" section of the manuscript.

Reviewer 2

Comment: *In this paper, "Fusing a Bayesian case velocity model with random forest for predicting COVID-19 in the U.S." by Watson et al. (2020), the authors propose an approach to forecasting mortality forecasts for the ongoing COVID-19 pandemic in the United States by fusing regression models with compartment models. This method is validated using a holdout sample of cases and deaths data, and used to make forecasts for future unobserved cases and deaths.*

I applaud the authors for their improvements and appreciate the detailed way they address the comments I made on the first draft. Overall I believe the paper has been significantly strengthened now lays out an appealing fusion of epidemiological dynamics and statistics / machine learning methodology. However, I still have a few reservations which I detail below.

Reply: Thank you for these comments.

Comment: *I am somewhat more appreciative of the merits of modeling the "velocity" as the authors define it. The main merit seems to be that, compared to modeling the daily case numbers, it can handle underreporting by rather looking at the proportional daily increase in cases. However, I am still not fully convinced that the velocity model is the best measure. The model assumes that the velocity has independent Gaussian errors with the variance decreasing linear in time, due to the velocity being inversely proportion to the current cumulative number of cases. I must say that this a bit clumsy to me. One idea I had was modeling the log-growth rate. Let $v(t)$ be the daily number of cases one day t. The log-growth rate is*

$$y'(t) = \log \frac{v(t)}{v(t-1)}.$$

This looks similar to the derivative of the cumulative case number, and like the velocity measure it is more robust to underreporting. However, with the growth rate it might not be necessary to shoehorn in the assumption of linearly decreasing variance.

Reply: The linear velocity model has indeed proved to be too restrictive, especially in light of the second wave that has occurred over the fall and winter of 2020. We have revised the velocity model, replacing the linear model with an autoregressive (AR) time series model with location specific parameters. The full details of the model specification may be found in section entitled "Bayesian Velocity Model for Forecasting Cases," which begins on page 4. The AR model better models the intermittent spikes or surges that have become characteristic of the pandemic in the United States. It also extrapolates forward in a less rigid manner, which provides more realistic trajectories. In addition it allows for an alternative derivation of the SIRD model transition function without solving for the value of the additional integration constant, which was required for the linear model. This is both conceptually cleaner and computationally expedient. The full details of the new derivation are included in supplement section S3.

Modeling the log growth rate is a potentially interesting alternative, but is a substantial departure from the velocity approach we have taken. In particular we prefer to continue modeling the velocity because it naturally smooths the variation in case growth, which may be quite pronounced.

Comment: *I agree with reviewer 1's comment no. 2 regarding the expression of the SIRD model. As the authors currently have it, it looks like subjects go into the R compartment before going to the D compartment. This is incoherent. Rather, as*

reviewer 1 says, the I compartment should branch out into R and D. The fact that R is unobserved is irrelevant to this fact.

Reply: We agree that requiring subjects to pass through the R compartment before entering D would be problematic. A typical parameterization of a model that allowed subjects to exit compartment I into R or D might look something like:

$$\begin{aligned}\frac{dS(t)}{dt} &= -\xi(t),\\ \frac{dI(t)}{dt} &= \xi(t) - \zeta I(t) - \theta I(t),\\ \frac{dR(t)}{dt} &= \zeta I(t),\\ \frac{dD(t)}{dt} &= \theta I(t).\end{aligned} \tag{1}$$

The transition parameters ζ and θ could of course be time dependent, i.e., $\zeta(t)$ and $\theta(t)$. We have chosen a slightly different parameterization, because we are not confident we can accurately estimate ζ, the rate at which infectious subjects recover, especially if it varies over time. We do have data on subjects moving into D, which we use to train the random forest death model. This still leaves us without a mechanism for moving subjects from I into R. We could either use a value for ζ (the rate at which subjects enter R from I) from the literature or for ρ in our parameterization, which is the rate at which subjects exit I for either R or D. We have chosen the latter, because we feel the evidence was stronger for this. So we are not moving subjects into R and then D, we are moving subjects out of I and then divvying them up between R and D. In essence we have split the R compartment of a traditional SIR model into R and D, with $\theta(t)$ defining the partition.

Comment: *The ρ parameter in your SIRD model should correspond to estimates of the time from infection to recovery or death rather than what is recommended by CDC; for instance, see Verity et al. (2020): https://www.thelancet.com/journals/laninf/article/PIIS1473-3099(20)30243-7/fulltext*

Reply: Individuals in compartment I are infectious cases, and we conceptualize the transition rate out of I as the rate at which an infectious individual who has tested positive (and therefore is a case) becomes not infectious either by recovering or dying. This is a simplification of the clinical reality in which many individuals are no longer infectious before they have fully recovered from their illness. We do not require individuals entering compartment R to be free of symptoms, only that they not be infectious.

Using onset of symptoms as a proxy for testing positive (i.e., moving from S to I), we set the mean for ρ^{-1} to be 10, based on Wölfel et al. estimating a less than 5% probability of isolating virus at 9.78 days after symptom onset (Wölfel, Roman, et al. "Virological assessment of hospitalized patients with COVID-2019." *Nature* 581.7809 (2020): 465-469.). We have updated the discussion of ρ to clarify this point. It now reads,

> Like a traditional SIR model, we let $\rho I(t)$ denote individuals exiting the infectious compartment, which corresponds to the $-\rho I(t)$ term in $dI(t)/dt$. Since individuals do not enter compartment I until they test positive, in our model ρ^{-1} is the length of time we expect an individual to remain infectious after testing positive. Using onset of symptoms as a proxy for testing positive, we sample ρ^{-1} independently for each run from

> a Gaussian distribution with mean 10 and standard deviation 1, based on Wölfel et al. estimating the probability of isolating virus dropping below 5% at 9.78 days after symptom onset.

Comment: *I believe there is an equal sign missing in line 224, which should read:* $dS_i(t)/dt = -du_i(t)/dt = -\xi_i(t)$

Reply: Thank you for pointing out this omission. We have corrected it in the revised manuscript.

Comment: *I don't expect the authors to do this, but I think one ultimate goal could be constructing a joint model combining all these components, i.e. the log-linear model for cases, the tree model for deaths, and the differential equation system. This model would be "fully Bayesian" if you swap in BART for random forests.*

Reply: A fully Bayesian model in which BART were substituted for the random forest death model is certainly appealing and a worthy objective of future work. We have modified our discussion of this point to specifically include mention BART as a Bayesian alternative to random forest. It now reads,

> Future methodological improvements could include integrating all the components of the model within a single Bayesian model by substituting Bayesian additive regression trees (BART) for the random forest death model. This would provide a posterior distribution for all parameters and forecasts.
